# Correlation between chromatin epigenetic-related lncRNA signature (CELncSig) and prognosis, immune microenvironment, and immunotherapy in non-small cell lung cancer

**DOI:** 10.1371/journal.pone.0286122

**Published:** 2023-05-24

**Authors:** Zhuolong Xiong, Zenglei Han, Weiyi Pan, Xiao Zhu, Caixin Liu

**Affiliations:** 1 Clinical Laboratory, The First Affiliated Hospital of Wannan Medical College (Yijishan Hospital of Wannan Medical College), Wuhu, China; 2 Computational Systems Biology Lab (CSBL), Institute of Bioinformatics, The Marine Biomedical Research Institute, Guangdong Medical University, Zhanjiang, China; 3 Department of Pathology, Qingdao Municipal Hospital, Qingdao, China; 4 Zhejiang Provincial People’s Hospital, People’s Hospital of Hangzhou Medical College, Hangzhou Medical College, Hangzhou, China; Xiangya Hospital Central South University, CHINA

## Abstract

Chromatin regulators drive cancer epigenetic changes, and lncRNA can play an important role in epigenetic changes as chromatin regulators. We used univariate Cox, LASSO, and multivariate Cox regression analysis to select epigenetic-associated lncRNA signatures. Twenty-five epigenetic-associated lncRNA signatures (CELncSig) were identified to establish the immune prognostic model. According to Kaplan-Meier analysis, the overall survival of the high-risk group was significantly lower than the low-risk group. Receiver operating characteristic (ROC) curves, C-index, survival curve, nomogram, and principal component analysis (PCA) were performed to validate the risk model. In GO/KEGG analysis, differentially expressed lncRNAs were correlated with the PI3K−Akt pathway, suggesting that they were highly associated with the metastasis of LUAD. Interestingly, in the immune escape analysis, the TIDE score was lower, and the possibility of immune dysfunction is also slighter in the high-risk group, which means they still have the potential to receive immunotherapy. And CELncsig is highly correlated with immune pathways T_cell_co-inhibition and Check-point. Also, the IMvigor210 cohort analysis indicated that our risk-scoring model has significant potential clinical application value in lung cancer immunotherapy. And we also screened out ten potential chemotherapy agents using the ‘pRRophetic’ package.

## Introduction

Today, lung cancer remains the leading cause of cancer-related mortality worldwide. A study examining 36 cancer types in 185 countries reported 2.2 million new lung cancer cases in 2020, making it the second most common cancer diagnosis. Concurrently, approximately 1.8 million individuals succumbed to lung cancer [1]. Non-small cell lung carcinoma (NSCLC) is a subtype of lung cancer with a poor prognosis, boasting a mere 19% five-year survival rate [2]. Lung adenocarcinoma (LUAD) is a subtype of NSCLC. Most LUAD primarily originates in the external area of the lung and tends to spread to lymph nodes and peripheral tissues [3]. Epigenetic changes to tumor-associated genes are common in LUAD, especially the inactivation of tumor suppressor genes [4].

Long non-coding RNAs (lncRNAs) are non-coding RNAs consisting of various RNAs that transcribe over 200 nucleotides in length and lack protein-coding potential. LncRNAs are more active than mRNA in biology, and surface lncRNAs play an essential role in biology [5]. One of the critical factors affecting cancer is the change in the cancer microenvironment, and the complex and dynamic environment around the tumor is called the tumor microenvironment (TME) [6]. There is considerable evidence that immune-related long non-coding RNAs play an essential role in the TME and have significant potential in immune regulation [7, 8]. LncRNAs are integral to the genomic regulatory network, particularly in transcriptomics. Compared to normal or adjacent cancerous tissues, numerous differentially expressed lncRNAs can be identified within tumor samples, which can be utilized for subtype classification and prognostic prediction [9]. Thus, investigating lncRNAs associated with epigenetic regulatory factors is essential for predicting tumor immunotherapy outcomes and developing prognostic models.

According to a 2018 publication, cancer arises from a malignant transformation driven by the accumulation of body-acquired inheritance and epigenetic aberrations [10]. In epigenetics, chromatin regulators (CRs) are usually divided into three parts, DNA methylation factors, histone modification, and chromatin remodeling factors. Changes in the expression of a single CR may affect the function of one or more components and potentially profoundly affect gene expression [11]. Presently, research on chromatin epigenetic-associated lncRNAs in lung cancer remains limited. As such, establishing a model of chromatin epigenetic-related lncRNA signature (CELncSig) is of great importance for predicting tumor prognosis.

**Fig 1:**
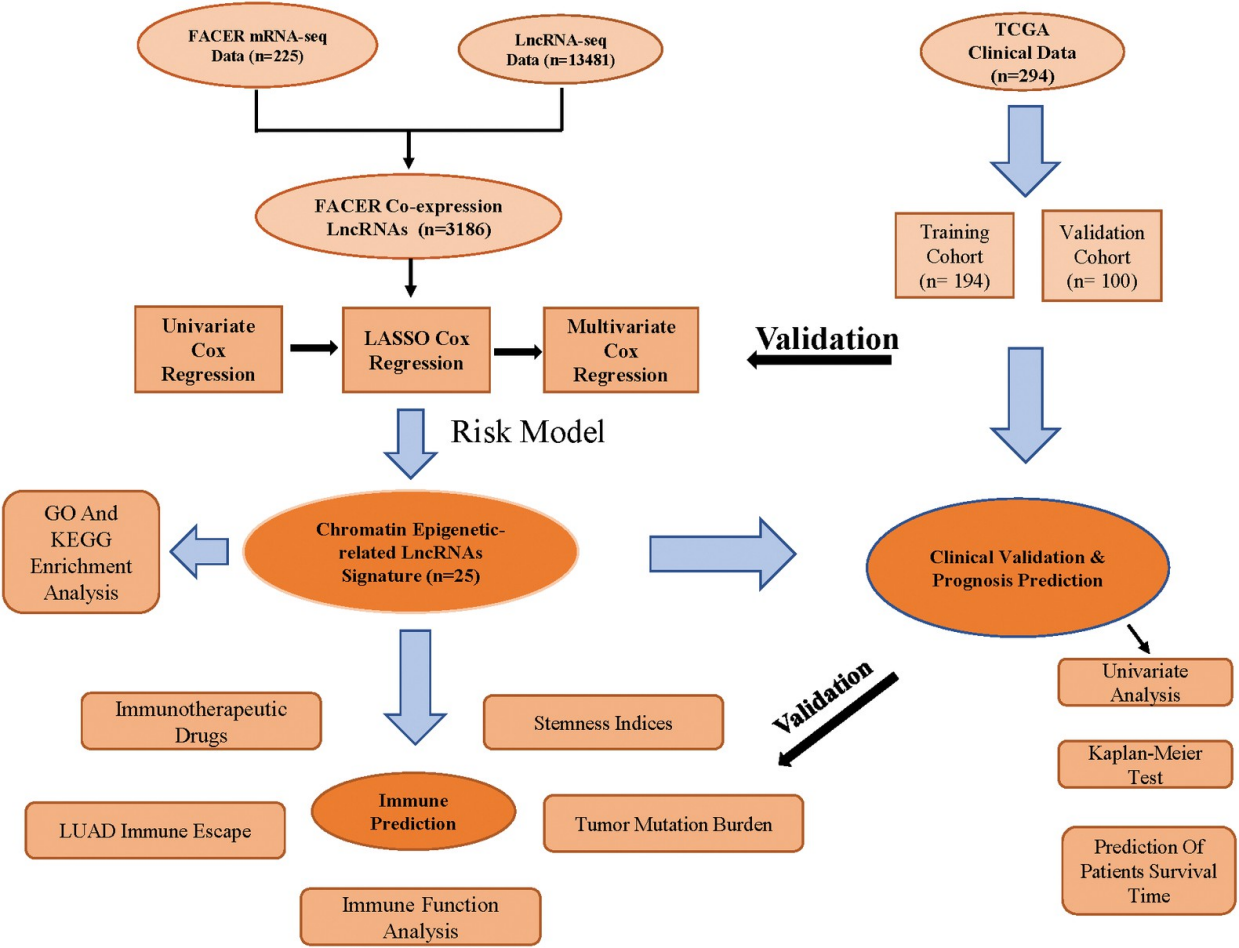
Flowchart of the research process. This includes data collection, identification of chromatin epigenetic-related lncRNA signatures, clinical data validation, prognostic prediction, and immunotherapy prediction.

In this study, we constructed the CELncSig prognostic model and explored its role in immune function and immunotherapy. The detailed methodology is illustrated in Fig 1.

## Materials and methods

### Ethical approval and consent to participate

The work was approved by the Guangdong Medical University Ethical Committee (YS2021159). Informed consent forms are not required for patient data extracted from public databases.

### The data source

We downloaded RNA sequence transcription data (FPKM), RNA transcription data (n = 19508), and lncRNA transcription data (n = 13481) from the TCGA website (https://portal.gdc.cancer.gov/). The Cancer Genome Atlas (TCGA) database is a comprehensive collection of genomic, epigenomic, and clinical data from cancer patients [12, 13]. In addition, we also obtained relevant clinical samples of lung cancer, including the following information: age, sex, tumor grade, TNM, stage, survival time, and survival status. “TNM” stands for primary tumor (T), regional lymph nodes (N), and distant metastasis (M). Clinical examples that did not meet the following conditions were removed: 1. non-primary tumor; 2. data unrelated to survival indicators (survival status and survival time) were not included. 302 LUAD clinical samples were formally included (S1 File). 8 LUAD samples were removed from the analysis due to missing expression data files. Finally, we selected 294 LUAD samples and randomly divided them into a training cohort (n = 194) and a validation cohort (n = 100). The correlation test of pathological features is shown in S1 Table in S3 File. Due to the retrospective design of the study and the anonymous analysis of patients’ data, obtaining informed consent was waived.

Epigenetic changes are driven by chromatin regulators (CRs). FACER (Functional Atlas of Chromatin Epigenetic Regulators) is an approach to prioritize the role of functional chromatin regulators in cancer. This method screened applicable CRs from 10,969 tumors of 33 cancers. Our genetic base is the sifting associated with lung cancer 225 functional CRs [10]. The 225 functional CRs in the gene set include 32 cancer-common CRs and 193 cancer-specific CRs (S2 File). These functional CRs play vital roles in histone modification, chromatin remodeling, and DNA methylation [10].

### Establishment of the risk model

The Pearson correlation analysis (| cor | > = 0.4 and P< 0.001) was used to extract the mRNA expression of chromatin epigenetic regulators in LUAD, and the lncRNAs co-expressed with gene sets were identified by the ’LIMMA’ package in R (version 4.1.3) and correlation test [14]. To find lncRNAs associated with prognosis, a univariate Cox regression analysis was performed (significance filter criteria P-value<0.05). LASSO regression analysis was used to further reduce the independent variables required by the model and prevent over-amplification of prognostic features. Multivariate Cox regression analysis was performed to establish a meaningful prognostic model. Finally, 25 selected CELncSigs were used to construct the immunodiagnostic lncRNA model (significance filtering criteria P-value<0.05) [15]. We created a method for calculating individual risk scores based on chromatin epigenetic associated lncRNA (CELncSig) expression as our risk scoring formula:

$$CELncSig=\sum_{i=1}^{n}{Coef}_{i}\times {expr}_{{lncRNA}_{n}}$$

Coef_i_ is the regression coefficient in multivariate Cox regression, and ${expr}_{{lncRNA}_{n}}$ is the expression level of lncRNA. Patients with high-risk scores had poorer expected survival [16, 17]. We used the median risk score as a cutoff to divide LUAD patients into high-risk and low-risk groups.

### The predictive ability of the CELncSig prognostic risk model and validation of the model

Meanwhile, we used a heatmap to represent the relationship between the model-related lncRNAs and the risk score. Subsequently, univariate and multivariate Cox regression analyses were performed using clinical-grade lung cancer data (age, sex, etc.). The purpose of the Cox analysis is to evaluate the impact of several factors on survival simultaneously.

In the evaluation of the model, we used ROC (Receiver operating characteristic) analysis to evaluate the quality of the patient’s clinically independent prognostic model. Furthermore, we used clinical C-index curves to further evaluate the quality of patient-independent prognostic models. Finally, we verify our model by using a nomogram and calibration curve. The nomogram can be applied to the graphic calculation of complex formulas with practical accuracy.

### Validation of clinical grouping data and assessment of patient differentiation

The ‘survival’ package in R (version 4.1.3) was used to verify the grouped data of clinical data. The clinical indicators included stage, age, gender, survival state (fustat), race, AJCC (The American Joint Committee on Cancer) stages, primary tumor (T), regional lymph nodes (N), and distant metastasis (M). Kaplan-Meier analysis was used to analyze the survival parameters of risk groups. Finally, we used three-dimensional principal component analysis (PCA) to analyze whether there was a differentiation between coding genes, non-coding genes, and all genes in the high and low-risk groups.

### GO/KEGG pathway enrichment analysis of the risk model

We brought the LUAD-related lncRNAs into the high/low-risk model and used the ‘LIMMA’ package in R (version 4.1.3) for differential expression analysis. We also used the ‘clusterProfiler’ package in R to perform GO (Gene Ontology) function annotation for 448 differentially expressed lncRNAs. FDR of 0.05 was considered statistically significant. We also used bar charts to specifically display differentiated lncRNAs related to cell components (CC), molecular functions (MF), and biological processes (BP). Meanwhile, we performed the KEGG (Kyoto Encyclopedia of Genes and Genomes Enrichment Analyses) pathway analysis.

### Analysis of immune function and tumor mutation burden in risk groups

Based on specific immune genes, immune gene function can be classified into 13 types of immune events [18, 19]. To determine the scores of high and low-risk groups in these 13 immune function pathways, we utilized single-sample gene enrichment analysis (ssGSEA) [19, 20]. Recent studies have established a close association between tumor mutation burden and the efficacy of anti-programmed cell death ligand 1 (anti-PD-L1) therapy in several types of cancer [21, 22].

Subsequently, we employed the ’LIMMA’ package in R (version 4.1.3) to analyze the difference in tumor mutation burden between the high and low-risk groups of LUAD. Furthermore, we utilized the ’survival’ package to evaluate the overall survival rate between groups with high and low tumor mutation burden.

### LUAD immune escape analysis and identification of potential therapeutic drugs

Tumor immune dysfunction and exclude files (TIDE) score from the TIDE website (http://tide.dfci.harvard.edu). The immune biomarker Interferon-gamma (IFNG) has been found to play a critical role in both innate and adaptive immune responses, while at the same time, T-cell-inflamed signature (Merck18) can contribute to T-cell dysfunction, demonstrating the important roles of a series of immune checkpoint biomarkers in cancer immune mechanisms [23, 24]. We compared the model high-low risk groups with eleven known tumor immune markers, including Microsatellite Instability Score (MSI) [25, 26], the cluster of differentiation 8 (CD8), the cluster of differentiation 274 (CD274) [26–28], Dysfunction, Exclusion, tumor-associated macrophages M2 (TAMM2) [29], Myeloid-derived suppressor cell (MDSC) [30], Cancer-associated fibroblasts (CAFs), Merck18, IFNG to analyze the role of high and low-risk groups in tumor immune escape and prediction model in the effect of immunotherapy [31].

After utilizing the ‘pRRophetic’ package to identify potential therapeutic agents for LUAD, we quantified the sensitivity of high and low-risk groups to chemical agents by using IC50 as a measure of drug inhibition [32]. The ‘pRRophetic’ package has the capability to predict clinical chemotherapeutic response through the utilization of tumor gene expression data [22].

### Prediction of immunotherapy effect and analysis of immunotherapy response of the model

IMvigor210 cohort data were downloaded from the website (https://clinicaltrials.gov/ct2/show/NCT02108652). Atezolizumab is an anti-PD-L1 antibody immunotherapy agent and can be used as a co-immune checkpoint inhibitor in the treatment of advanced or metastatic bladder cancer [19]. Programmed cell death ligand 1 (PD-L1) related genes are also expressed in LUAD. Therefore, the immunotherapy result of the IMvigor210 cohort can be used to verify the immunotherapy prediction ability of the risk model [19, 33]. We combined lncRNAs from the LASSO regression analysis with genes from the IMvigor210 cohort to create a multivariate Cox risk model. And plot the survival curves of high and low-risk groups. ROC curves were used to evaluate the model’s predictive ability.

Stemness Indices is an index that describes the similarity of cancer cells to stem cells and can be used as a prognostic indicator to predict the risk of tumor recurrence and guide treatment [34]. By using a one-class logistic regression (OCLR) algorithm to train the tumor stem cell (ESC, embryonic stem cell; iPSC, induced pluripotent stem cell) classes and their differentiated ectoderm, mesoderm, and endoderm progenitors, then applied to The Cancer Genome Atlas (TCGA) dataset to calculate the mRNA gene expression-based stemness index (mRNAsi) [35]. Our index of mRNA expression of lung cancer stem cells is based on the available data [34]. We combined lung cancer clinical data and lung cancer stem cell data and used the ‘survival’ package in R (version 4.1.3) for survival analysis of lung cancer mRNAsi. Finally, we further studied the clinical relevance of mRNAsi.

## Result

### Identification of co-expressed lncRNAs in FACER gene sets

We extracted 225 Facer-related genes from the database and extracted the mRNA expression levels of the gene sets. Then, we further extracted 3186 lncRNAs co-expressed with the gene set by Pearson correlation analysis (corFilter = 0.4 and pvalueFilter = 0.001). We randomly divided all selective patients (n = 294) into a training cohort (n = 194) and a validation cohort (n = 100). To verify our grouping, we analyzed the relationship between patients’ randomization and P value > 0.05, indicating no significant difference between the two groups and that the grouping results are promising (S1 Table in S3 File).

### Establishment of chromatin epigenetic-related lncRNAs prognostic model

We further combined gene-set-related lncRNAs and patient survival data, conducted a univariate Cox analysis on gene-set-related lncRNAs, and identified 206 lncRNAs associated with cancer prognosis and survival. To reduce the number of independent variables, the LASSO regression analysis was conducted first, then the multivariate Cox analysis. Finally, we identified 25 lncRNAs significantly associated with lung cancer prognosis (S2 Table in S3 File).

### Clinical assessment using risk models

**Fig 2:**
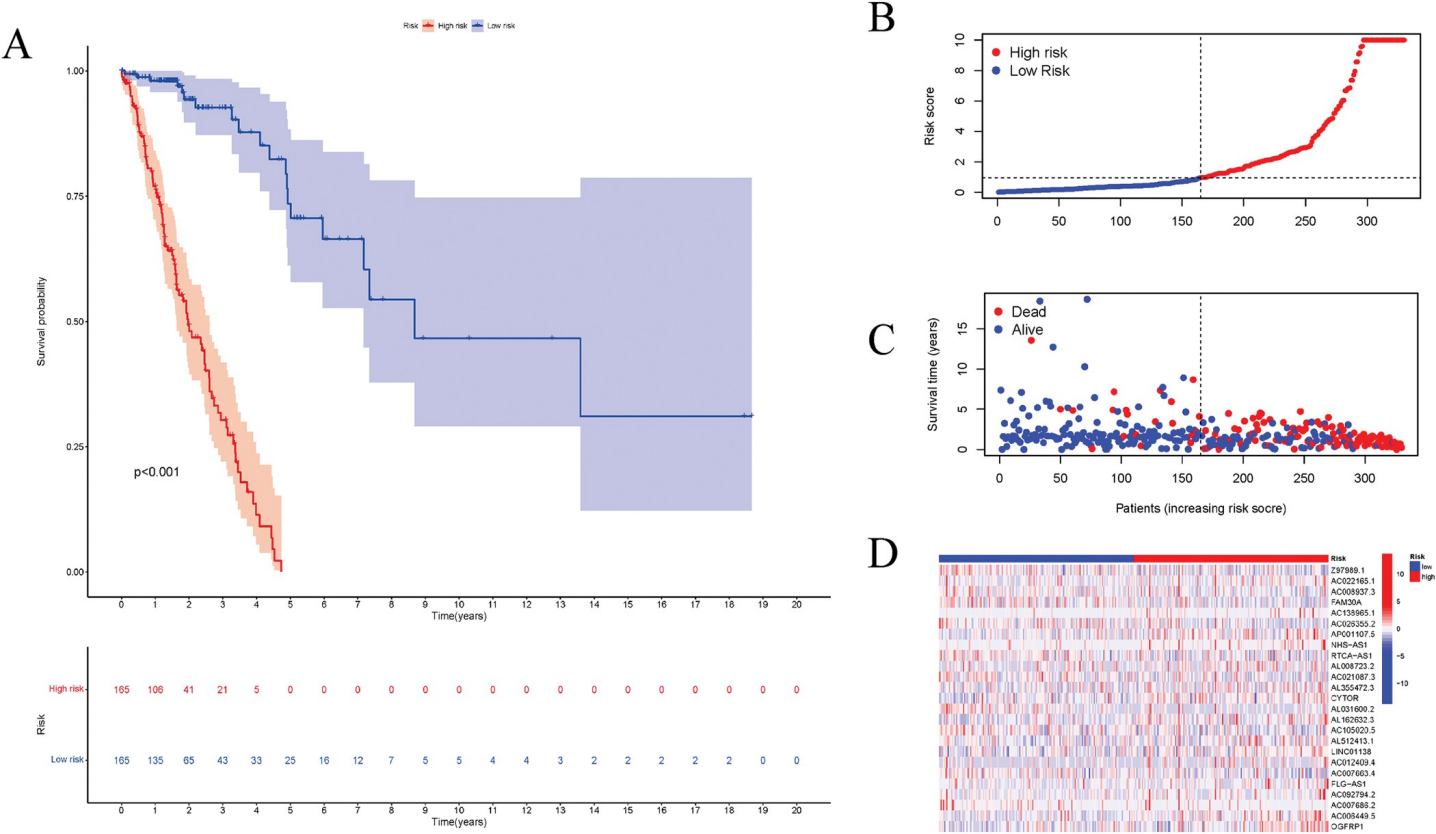
Prognostic prediction based on the identified lncRNA signature (CELncSig). (A) Patients in the low-risk group showed longer overall survival (OS) in the Kaplan-Meier analysis. (B, C) Survival status and risk scores for each sample. (D) Heatmap shows the expression of CELncSigs, with blue indicating low expression and red indicating high expression.

We categorized the patients into high- and low-risk groups according to the median risk score and compared the impact of differential expression of lncRNAs on overall survival rate. Patients in the high-risk group had significantly lower overall survival rates than those in the low-risk group (P< 0.001) (Fig 2). The high risk of LUAD was associated with AC138965.1 (HR = 3.41, 95% CI: 1.95–5.98, P<0.01) and AL162632.3 (HR = 3.49, 95% CI: 1.30–9.35, P = 0.01). Moreover, AC007686.2 was linked to a low risk of LUAD (HR = 0.16, 95% CI: 0.03–0.75, P = 0.002), which is protective in LUAD patients (Fig 2).

In univariate Cox analysis of clinical indicators, we identified that stage (HR = 1.552, 95% confidence interval: 1.303–1.849, P< 0.001), T (HR = 1.481, 95%CI: 1.162–1.888, P = 0.001), N (HR = 1.827, 95%CI: 1.454–2.296, p< 0.001) were associated with the high-risk subgroup, while no significant results were found in multivariate Cox analysis (S1 Fig in S3 File). Furthermore, our results showed that patients with higher risk scores had a greater likelihood of death, and the risk of death increased from tumor stage I to stage III. Additionally, male patients had a higher risk of death (S2 Fig in S3 File) (P<0.05).

### Evaluation and validation of the risk model

The C-index curve demonstrated that values of various clinical indicators such as risk score, stage, regional lymph nodes (N), and primary tumor (T) were greater than 0.5, which further verified the excellent predictive ability of the model (S3 Fig in S3 File). Subsequent calibration curve verification revealed that the predicted values of patients’ 1-year, 3-year, and 5-year survival rates were close to the ideal curve, indicating that the model plays a crucial role in predicting patients’ prognosis (S3 Fig in S3 File). The ROC curve analysis indicated that most indicators in the model, such as risk, age, stage, T stage, M stage, and N stage, could serve as short- and long-term prediction indicators, except for age, gender, and race (S3 Fig in S3 File). The model has good accuracy in prediction in the short and long term (1-year AUC = 0.797, 3-year AUC = 0.813, 5-year AUC = 0.830) (S3 Fig in S3 File).

**Fig 3:**
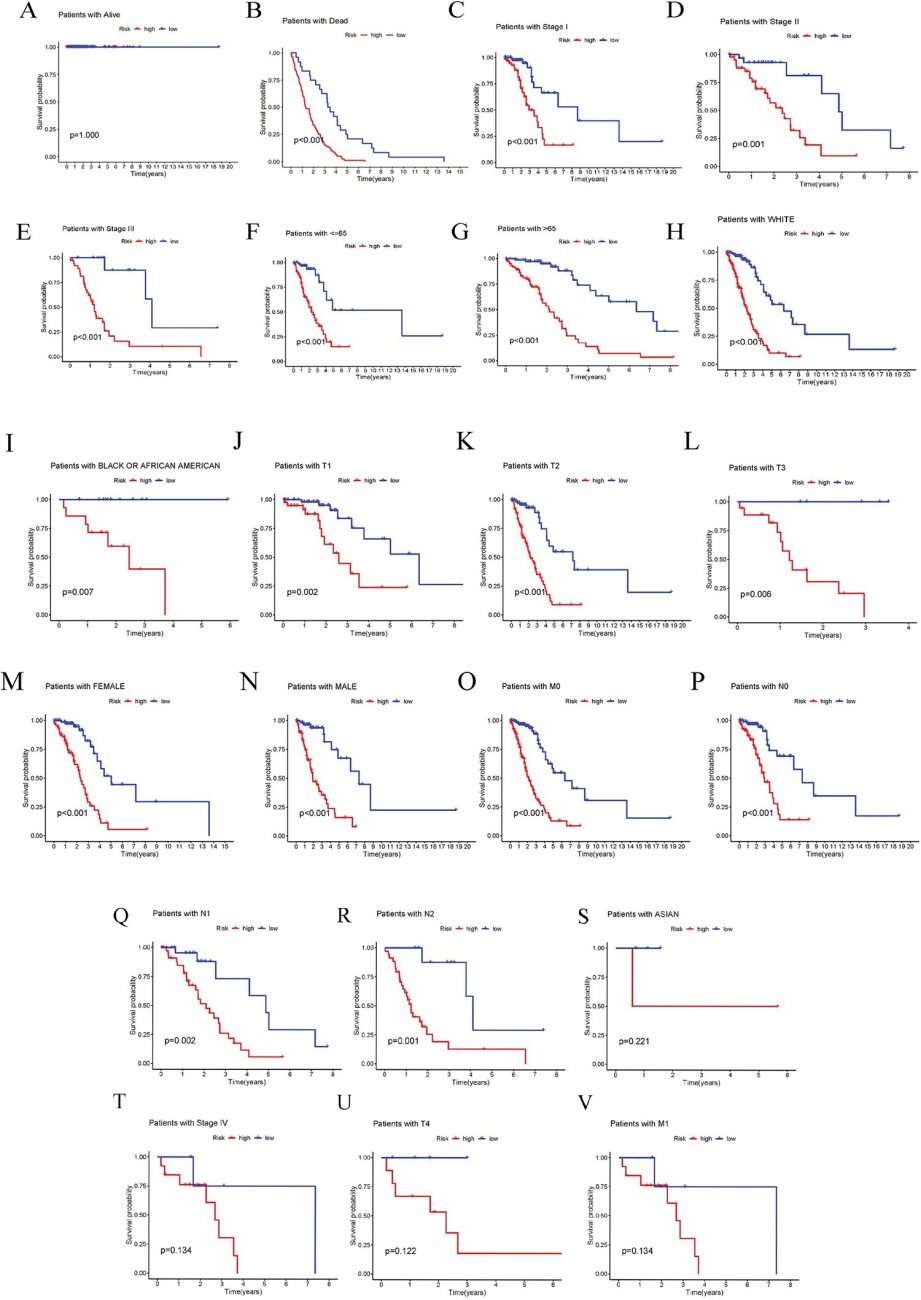
Validation of the prognostic prediction. (A, B) Calibration curves show the consistency between predicted and observed OS. (C-N) Kaplan-Meier OS curves of subgroups stratified by clinical factors, including AJCC stages (C-E), age (F, G), race (H, I), T stage (J-L), gender (M, N), M stage (O), and N stage (P-R).

Subsequently, the Kaplan-Meier analysis showed that the overall survival of the high-risk group was significantly lower than that of low-risk patients over time (P<0.05) (Fig 3). Additionally, compared to patients with a low-risk score, patients with stage I-III, patients of all ages, patients with white or black or African American race, patients at T1-T3, male and female patients, patients with M0, and patients with N0-N2 had significantly shorter overall survival (Fig 3). Finally, the results of the principal component analysis indicate good differentiation (S4 Fig in S3 File).

### GO/KEGG pathway enrichment analysis

**Fig 4:**
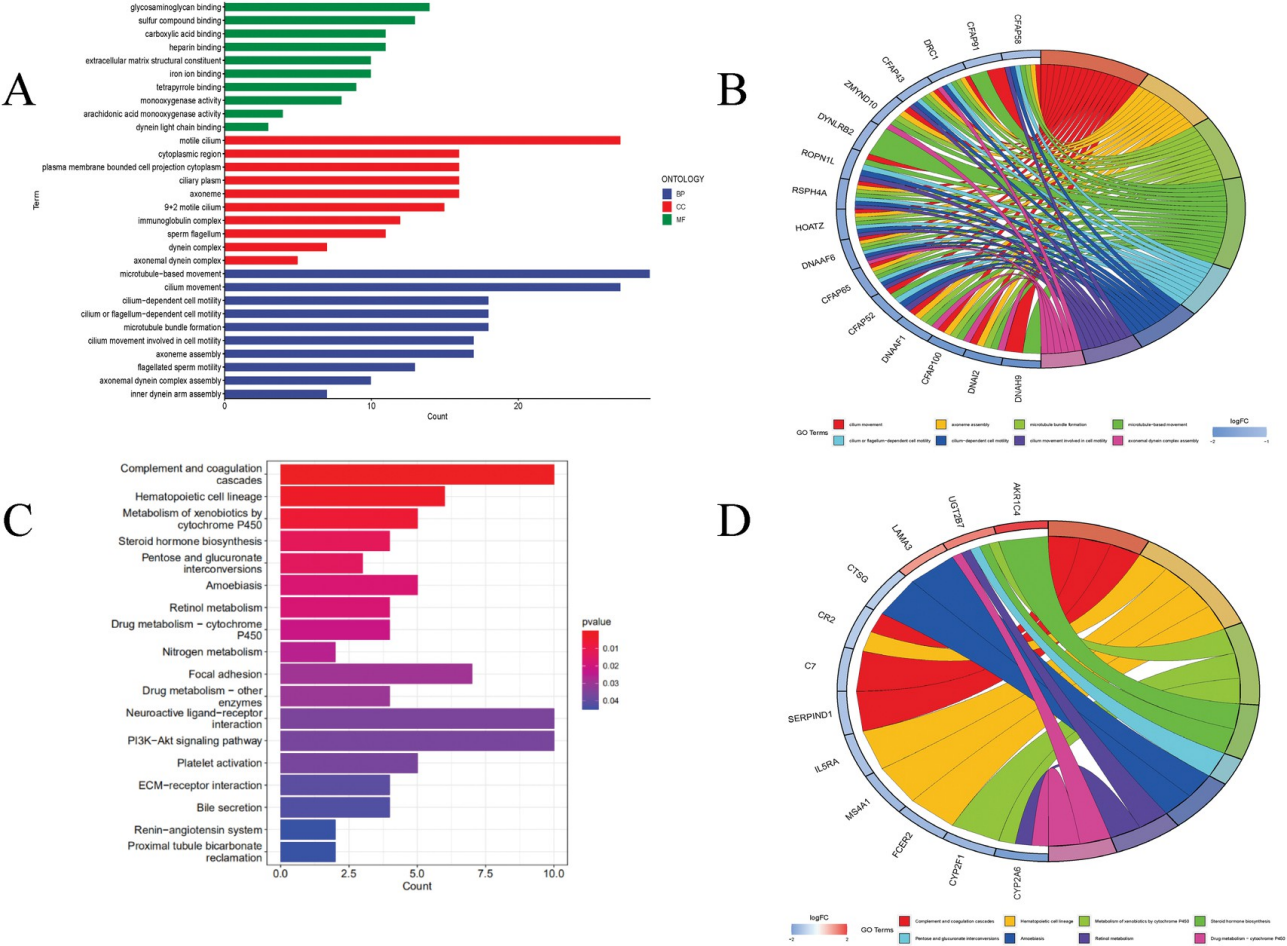
GO and KEGG pathway analysis of the differentially expressed lncRNAs between high- and low-risk groups. (A, B) GO enrichment analysis. (C, D) KEGG enrichment analysis.

We found a significantly different in the high-low risk group (P< 0.05), ‘clusterProfiler’ in R was used to perform functional enrichment analysis of differentially expressed lncRNA genes in the high-low tumor risk model by GO and KEGG. GO enrichment results showed that most of the differentially expressed lncRNAs were related to the movement of microtubules, including DYNLRB2, CFAP91, and ZMYND10. And most of the differentially expressed lncRNAs were related to the movement of motile cilia, including DNAH9, CFAP91, etc. Most differentially expressed lncRNAs are related to glycosaminoglycan binding, including ZMYND10 and DNAI2 (Fig 4). KEGG functional enrichment analysis showed that most of the differentially expressed lncRNAs were associated with replenishment and coagulation cascades (P< 0.01), such as SERPIND1, C7, CR2, followed by the interaction with neuroactive ligand-receptor and PI3K-Akt signaling pathway (P< 0.05) (Fig 4).

### Analysis of immune function and TMB

Based on our immune function analysis, in the same immune cluster, Cylolytic_activity, Inflammation-promoting, T_cell_co-inhibition, Check-point, and High expression of t_cell_co-stimulation genes were associated with the low-risk group (P< 0.05), which can be verified in the results of the validation cohort and all cohort (S5 Fig in S3 File). In the subsequent tumor mutation burden (TMB) analysis, our results showed that the high/low-risk group was not correlated with the TMB. However, we found that in the survival analysis, the high-risk group with the high-tumor mutational burden group had a significantly worse prognosis compared with the low-risk group with low TMB. The survival probability was higher in the low-risk group with a high TMB and in the low-risk group with a low TMB (P< 0.001) (S5 Fig in S3 File).

### Immune escape analysis of risk model

**Fig 5:**
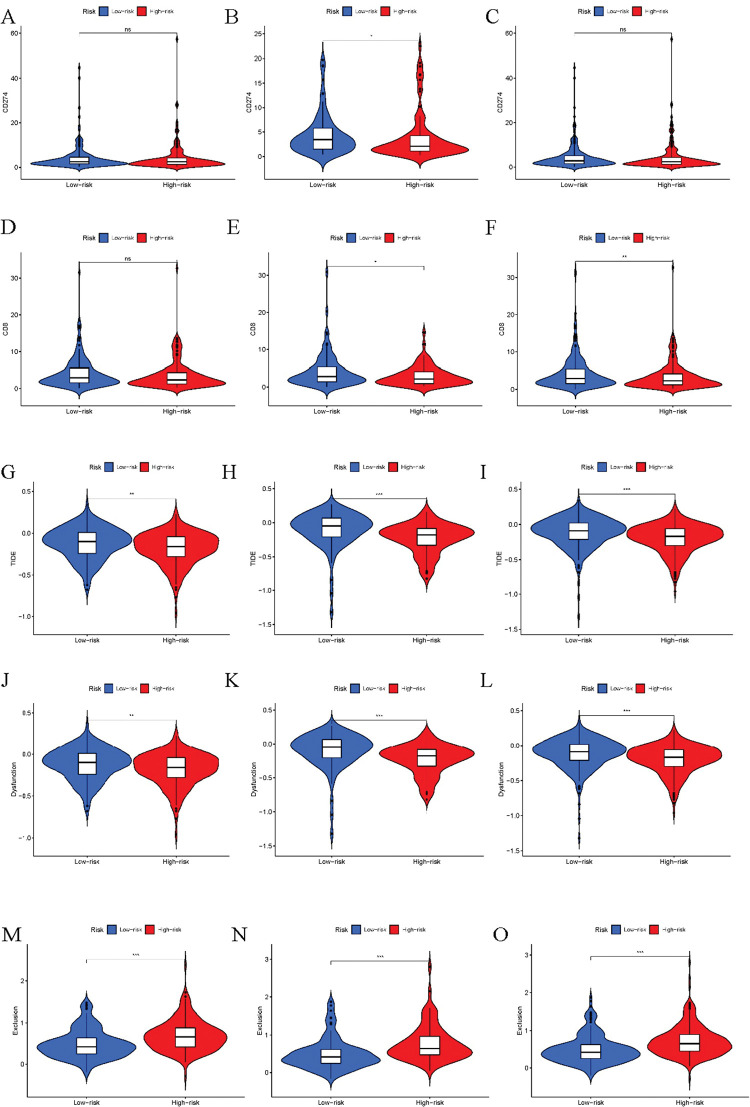
Tumor immune escape analysis of the cohorts. Comparison of CD274, CD8, TIDE, Dysfunction, and Exclusion scores between high- and low-risk groups in the (A-E) training, (F-J) validation, and (K-O) all cohorts. "*" means P value<0.05, "**" means P value<0.01, "***" means P value<0.001. "ns" means no significance.

To study the immune escape mechanism of LUAD, we focused on evaluating 11 markers related to LUAD immunogenicity. We first focused on mechanisms of internal tumor escape. There was no significant difference between immune markers CD274 and CD8 in the two groups (Fig 5). Interestingly, results showed that the TIDE score in the high-risk group was significantly lower, indicating the tumor immune escape potential was smaller (Fig 5). In addition, compared with the low-risk group, the high-risk group is more prone to immune rejection, but the possibility of immune dysfunction is lower (Fig 5).

**Fig 6:**
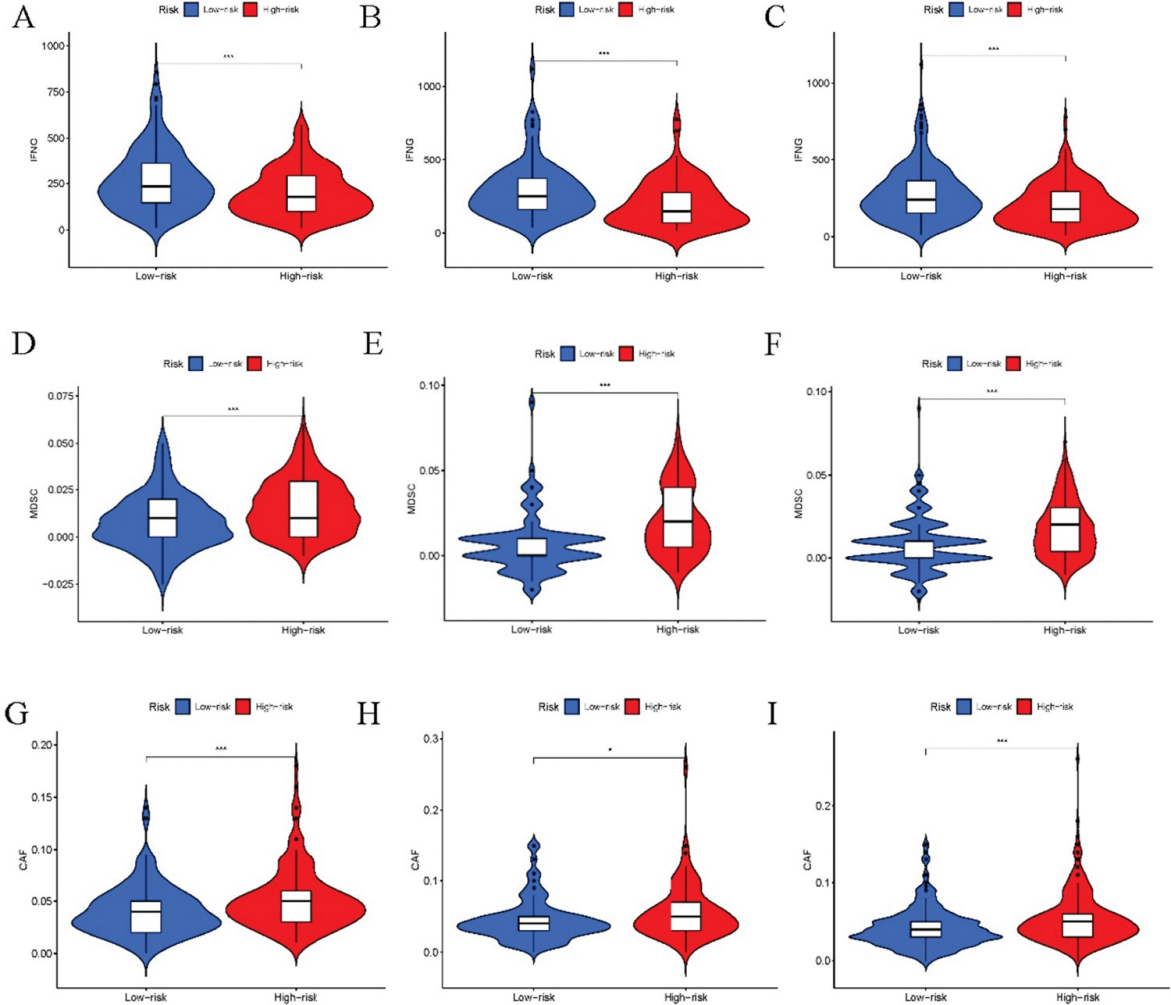
Tumor immune escape analysis of the cohorts. Comparison of IFNG, MDSC, and CAFs scores between high- and low-risk groups in the training, validation, and all cohorts. "*" means that P value<0.05, "**" means that P value<0.01, "***" means that P value<0.001. "ns" means no significance.

We then investigated the mechanisms of external tumor escape. Interestingly, interferon scores were higher in the low-risk group, prompting an increase in interferon may be associated with the low risk of LUAD (Fig 6). However, MDSC and CAFs (Fig 6) were associated with the high risk of LUAD.

### Screening of potential chemotherapy agents

**Fig 7:**
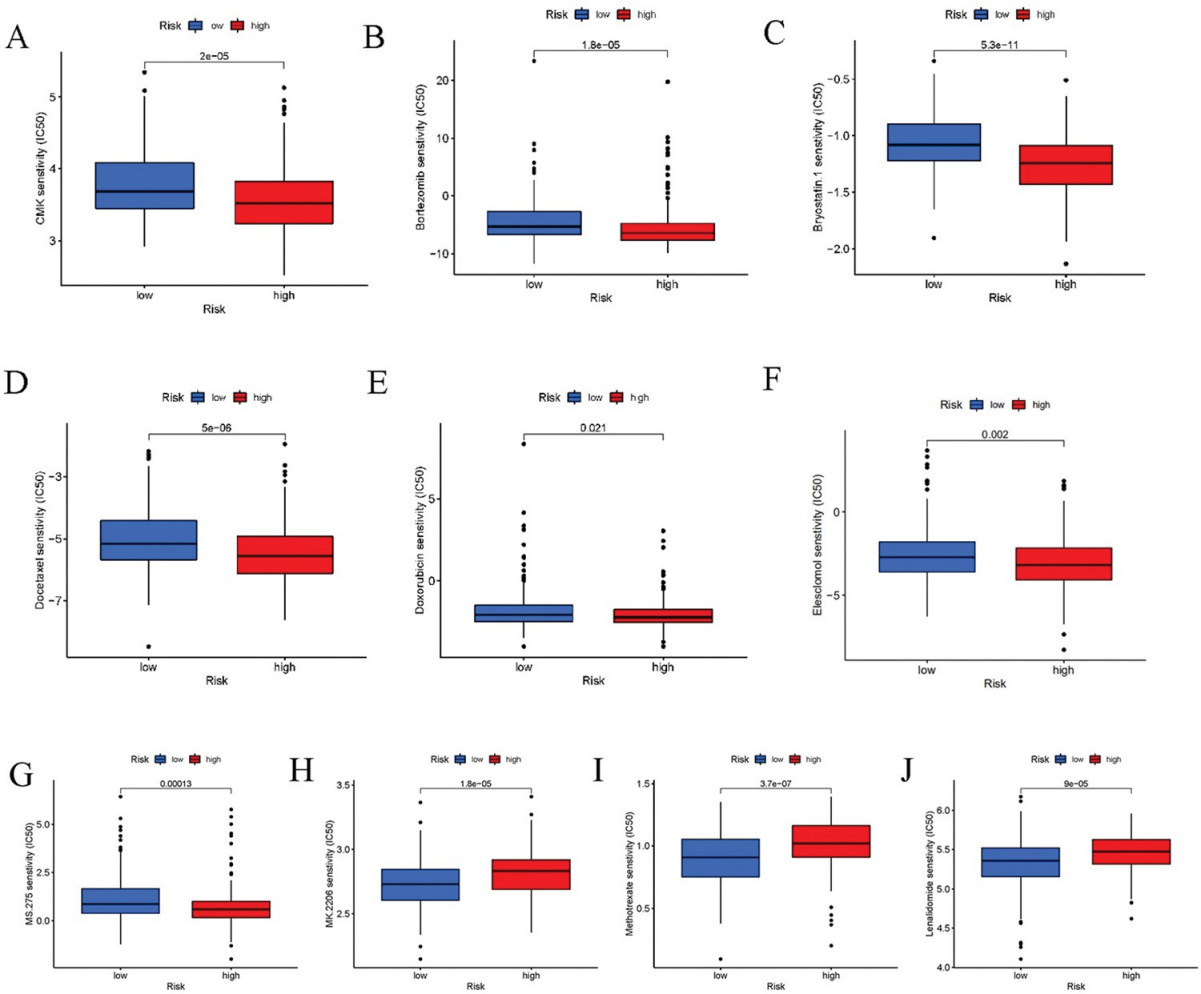
Prediction of differential chemotherapy drug sensitivity between high- and low-Risk groups. CMK, Bortezomib, Bryostatin 1, Docetaxel, Doxorubicin, Elesclomol, MS275, MK2206, Methotrexate, and Lenalidomide showed higher sensitivity in the low-risk group. CMK, Bortezomib, Bryostatin 1, Docetaxel, Doxorubicin, Elesclomol, and MS275 showed higher sensitivity in the high-risk group.

We then used ‘pRRophetic’ in R to screen for chemical drugs and validated our results with the validation cohort. Our results showed CMK, Bortezomib, and Bryostatin.1, Docetaxel, Doxorubicin, Elesclomol, MS275, Mk2206, Methotrexate, and Lenalidomide significantly differ between the two risk groups (Fig 7). Among them are CMK, Bortezomib, and Bryostatin.1, Docetaxel, Doxorubicin, Elesclomol, and MS275 showed higher sensitivity in high-risk groups, suggesting that these drugs have good efficacy in the treatment of the high-risk group. Mk2206, Methotrexate (Fig 7), and Lenalidomide were more sensitive in the low-risk group, and these results were replicated in the validation cohort and all cohorts (P<0.05).

### Predicting the effect of risk models on immunotherapy

Our analysis of immunotherapy response revealed significant differences in the risk scores of LASSO regression target genes among lung cancer patients with different responses to bladder cancer immunotherapy (P<0.05). The result indicates that the immunotherapy drug Atezolizumab has an excellent therapeutic effect in our CELncSig model (S6 Fig in S3 File).

There was no significant difference in LASSO regression target gene expression between the high and low-risk IMvigor210 cohort in LUAD patients with bladder cancer, indicating the prognostic effect of the IMvigor210 model was poor. However, we found that the target gene of LASSO regression in lung cancer patients predicted better three-year and five-year survival in the IMvigor210 cohort (AUC >0.5) (S6 Fig in S3 File).

### Stemness indices analysis

The analysis of stem cell indices revealed no significant effect of high or low stem cell indices on overall survival (S7 Fig in S3 File). However, there was a significant difference in the stem cell indices between lung cancer tissues and normal tissues (P<0.05). Further analysis demonstrated a significant correlation between clinical indicators and mRNAsi (S7 Fig in S3 File).

## Discussion

Over the past decades, various techniques have been employed to treat NSCLC, including surgery, chemotherapy, radiotherapy, targeted therapy, and immunotherapy. In immunotherapy, standard protocols emphasize immune checkpoint inhibitors, such as Keytruda and Opdivo, which target PD-1 or PD-L1 to activate the patient’s immune system [36]. However, immunotherapy is not suitable for all patients, and the effectiveness of immunotherapy depends on multiple factors, for example, the infiltrating of immune cells, the expression level of immune checkpoint genes, and somatic mutation status [37]. Therefore, it is crucial to develop effective immune characteristic models based on chromatin epigenetic regulator-related lncRNAs to improve the prognosis and assist clinicians and researchers in determining the appropriate immunotherapy approach.

CRs are essential to epigenetics, and tumor mutations are closely related to epigenetic processes. Our study demonstrates that the "CELncSig" chromatin epigenetic-related lncRNAs prognostic model is an independent prognostic factor for lung cancer patients. Through rigorous statistical analyses, we identified 25 lncRNAs significantly associated with lung cancer prognosis. The risk model, which incorporated these lncRNAs, effectively stratified patients into high- and low-risk groups, with the high-risk group exhibiting significantly lower overall survival. Univariate Cox analysis revealed that clinical indicators, such as stage, T stage, and N stage, were associated with the high-risk subgroup. Furthermore, the model demonstrated excellent predictive ability, as evidenced by C-index values, calibration curve verification, and ROC curve analysis. Kaplan-Meier analysis and principal component analysis further validated the model’s performance. Thus, the "CELncSig" risk model is a valuable tool in predicting prognosis for lung cancer patients and could potentially guide personalized treatment strategies to improve patient outcomes. An inaccurate prognostic staging system of lung cancer negatively impacts patient prognosis [38]. Therefore, physicians can estimate patients’ overall survival using the nomogram.

And AL162632.3 has the highest HR (HR = 3.49) in our model. A study has found that biomarker based on AL162632.3 may relate to immune functions such as human leukocyte antigen (HLA) and Type_II_IFN_Reponse [39]. And previous studies have demonstrated the crucial role of AL162632.3 in the cuproptosis model, ferroptosis model, and molecular chaperone model, all of which are based on LUAD samples [39–41]. This reveals the diverse functions of AL162632.3 in LUAD. Combining our research, we believe that AL162632.3 plays a vital role in LUAD, from the epigenetic level to the molecular regulation level of tumor growth, and is highly correlated with tumor cell death induction. Therefore, AL162632.3 may serve as an important biological marker and a promising therapeutic target for immunotherapy and chemotherapy.

To further study the role of differentially expressed lncRNAs in tumor, we discovered through GO enrichment analysis that most differentially expressed lncRNAs were related to microtubule movement, including DYNLRB2, CFAP91, and ZMYND10. KEGG enrichment analysis revealed that differentially expressed lncRNAs were related to the PI3K−Akt signaling pathway. India and Kinetochore Associated Complex Subunit 3 may facilitate LUAD metastasis by activating the PI3K−Akt signaling pathway, suggesting that PI3K−Akt has significant research value [42].

We examined the immune function of the prognostic model and found that Cylolytic_activity, Inflammation-promoting, T_cell_co-inhibition, Check-point, and T_cell_co-stimulation high expression of immune function-related genes were associated with low tumor risk. The relationship between Checkpoint and the risk model indicated that CElncSig might serve as a novel immunotherapy biomarker since various immune therapies have targeted the immune checkpoint of tumors [43].

Subsequently, we explored the mechanisms of tumor immune escape, focusing initially on the internal escape mechanisms of tumors. These escape mechanisms primarily include antigen-presenting ability inside a tumor, the expression of immune checkpoints (ICPs), and tumor immunogenicity [31]. Intriguingly, our results contradicted expectations, as the TIDE score was lower in the high-risk group, indicating less tumor immune escape potential. Additionally, the likelihood of immune dysfunction was also lower in the high-risk group. These findings may explain why immune escape did not occur in the high-risk group, suggesting that this group still has the potential to benefit from immunotherapy.

Relevant studies indicate that the external immune escape mechanism primarily comprises four aspects: leucocyte deficiency, a large number of immunosuppressive cells, a high concentration of immunosuppressive cytokines, and increased fibroblasts [31]. Interferon (IFN-γ) plays a multifaceted role in regulating the microenvironment of LUAD. However, interferon scores were lower in the high-risk group, suggesting that a decrease in interferon may be associated with an elevated risk of lung cancer [44]. We hypothesize that an increase in interferon contributes to immune escape in the high-risk lung cancer group.

We identified CMK, Bortezomib, and Bryostatin.1, Docetaxel, Doxorubicin, Elesclomol, and MS275 as potential therapeutic drugs for the high-risk group, and Mk2206, Methotrexate, and Lenalidomide as potential treatments for the low-risk group.

Understanding the differential response to Atezolizumab treatment in high and low-risk groups will enhance our comprehension of the role of risk scores in lung cancer. The results demonstrated that high and low-risk groups significantly differed in treating bladder cancer with Atezolizumab (P <0.05), indicating that our risk-scoring model holds considerable potential for clinical application in cancer immunotherapy.

The mRNA stemness index (mRNAsi) of lung cancer tissue was highly different from the normal tissue, indicating the potential of mRNAsi in clinical classification. Also, mRNAsi is closely associated with various clinical characteristics. Prior studies suggest that altered expression of mRNAsi in tissue can serve as a biomarker for identifying differentially expressed genes in cancer prognosis research [35, 45].

We acknowledge that our study has certain limitations, such as the need to better integrate our model with clinical indicators. Therefore, more clinical data is required to enhance our prognostic model.

In conclusion, we have established an immune prognostic model based on chromatin epigenetic-associated lncRNAs signature, which plays a vital role in predicting the overall survival of patients. The LUAD subgroups, differentiated according to the prognostic model, display distinct clinical, tumor immune escape, and biological function heterogeneity. The model can be utilized to improve patient prognosis, assess lung cancer stages, and screen chemotherapeutic drugs for lung cancer, offering significant clinical application value.

## Supporting information

S1 File (XLSX)

S2 File (XLSX)

S3 File Contains all the supporting figures and tables. (DOCX)
