# Supplementary material for: Correlation between chromatin epigenetic-related lncRNA signature (CELncSig) and prognosis, immune microenvironment, and immunotherapy in non-small cell lung cancer
Source: PLoS One. 2023 May 24;18(5):e0286122. doi: 10.1371/journal.pone.0286122 (PMC10208461; doi:10.1371/journal.pone.0286122)
Supplement: S3 File — (DOCX) [file pone.0286122.s003.docx]

**Supplementary materials**

**Abbreviations…………………………………………………………………………2**

**Figure S1………………………………………………………………………………3**

**Figure S2………………………………………………………………………………5**

**Figure S3………………………………………………………………………………7**

**Figure S4………………………………………………………………………………9**

**Figure S5……………………………………………………………………………..10**

**Figure S6……………………………………………………………………………..12**

**Figure S7……………………………………………………………………………..14**

**Table S1………………………………………………………………………………16**

**Table S2………………………………………………………………………………18**

**Abbreviations**

AUC, the area under the receiver operating characteristics;

GO, Gene ontology;

KEGG, Kyoto encyclopedia of genes and genomes;

LUAD, lung adenocarcinoma;

Pd-L1, Programmed cell death ligand 1;

ROC, receiver operating characteristic;

TCGA, The cancer genome atlas


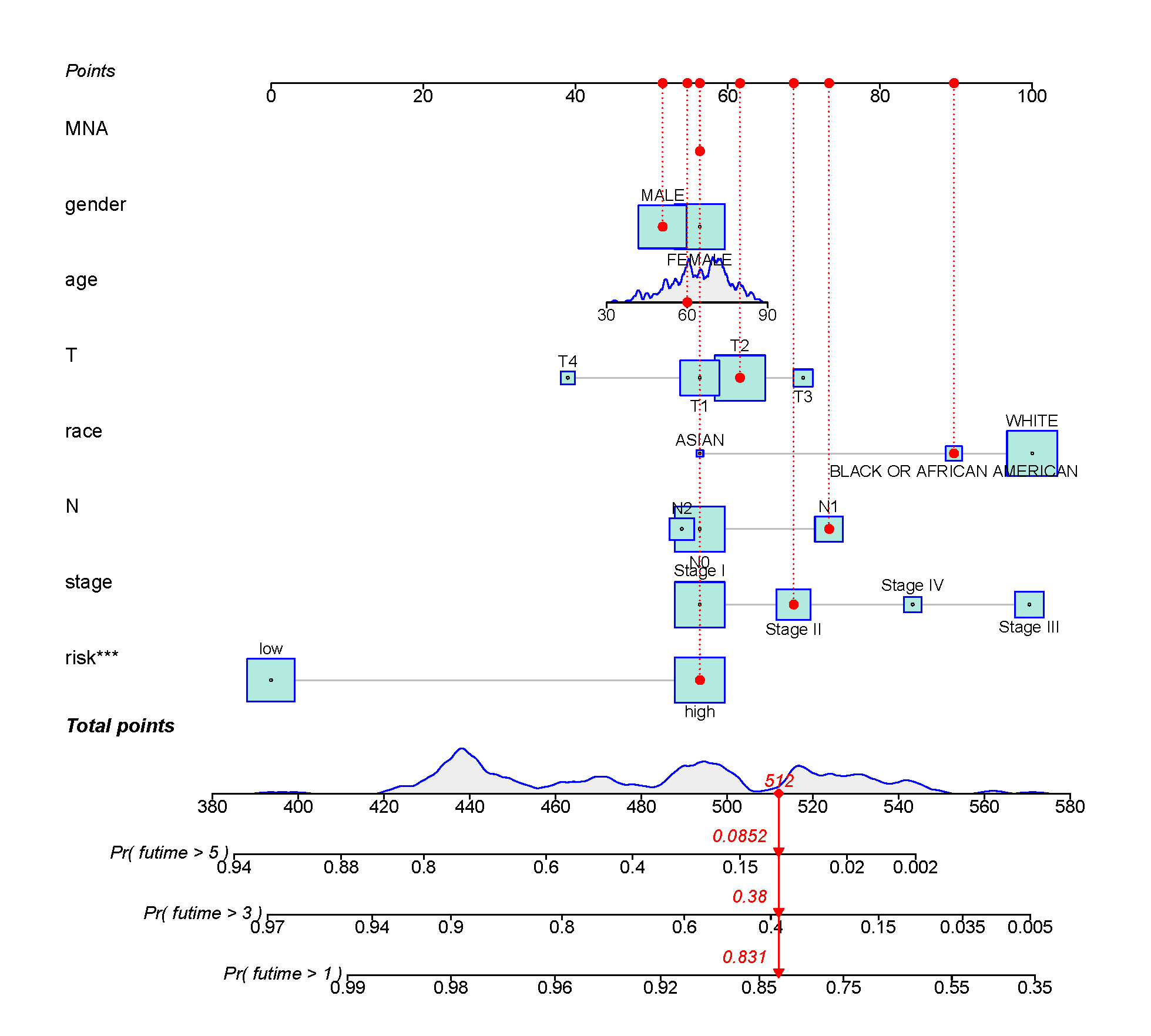


A

B


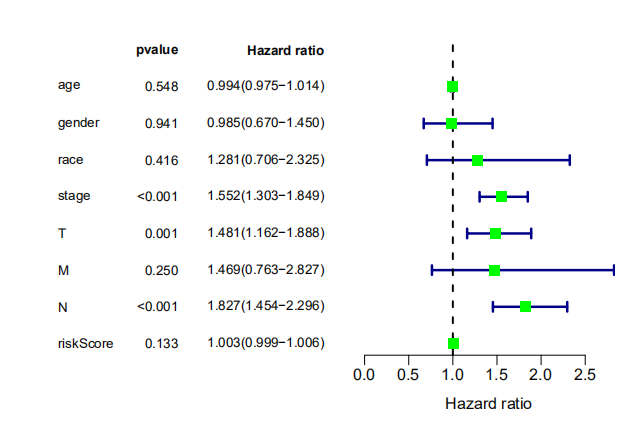


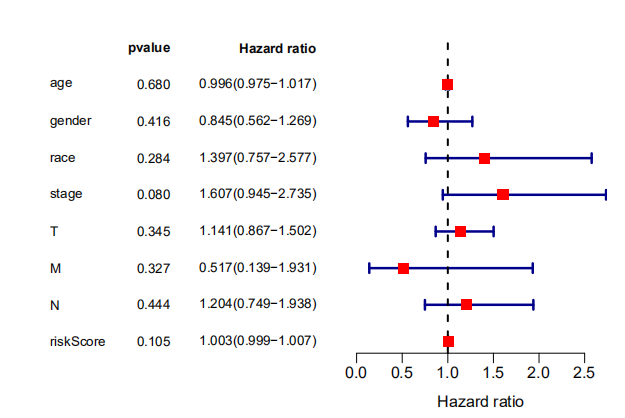


C

**Fig S1. A nomogram to predict the survival rate of LUAD.** The ordinate indicates each clinical index (A). "***" indicates P-value <0.001. The clinical indicators are used to draw vertical lines, and the score of all variables is calculated according to the patient's score on the abscissa to predict the patient's 1-year, 3-year, and 5-year survival probability (A). In the univariate Cox analysis, we found that Stage (HR=1.552, 95% CI: 1.303-1.849, p<0.001), T (HR=1.481, 95% CI: 1.162-1.888, p=0.001), and N (HR=1.827, 95% CI: 1.454-2.296, p< 0.001) were associated with high group. No clinical indicators associated with high-risk group were found in the multivariate Cox analysis (B, C).


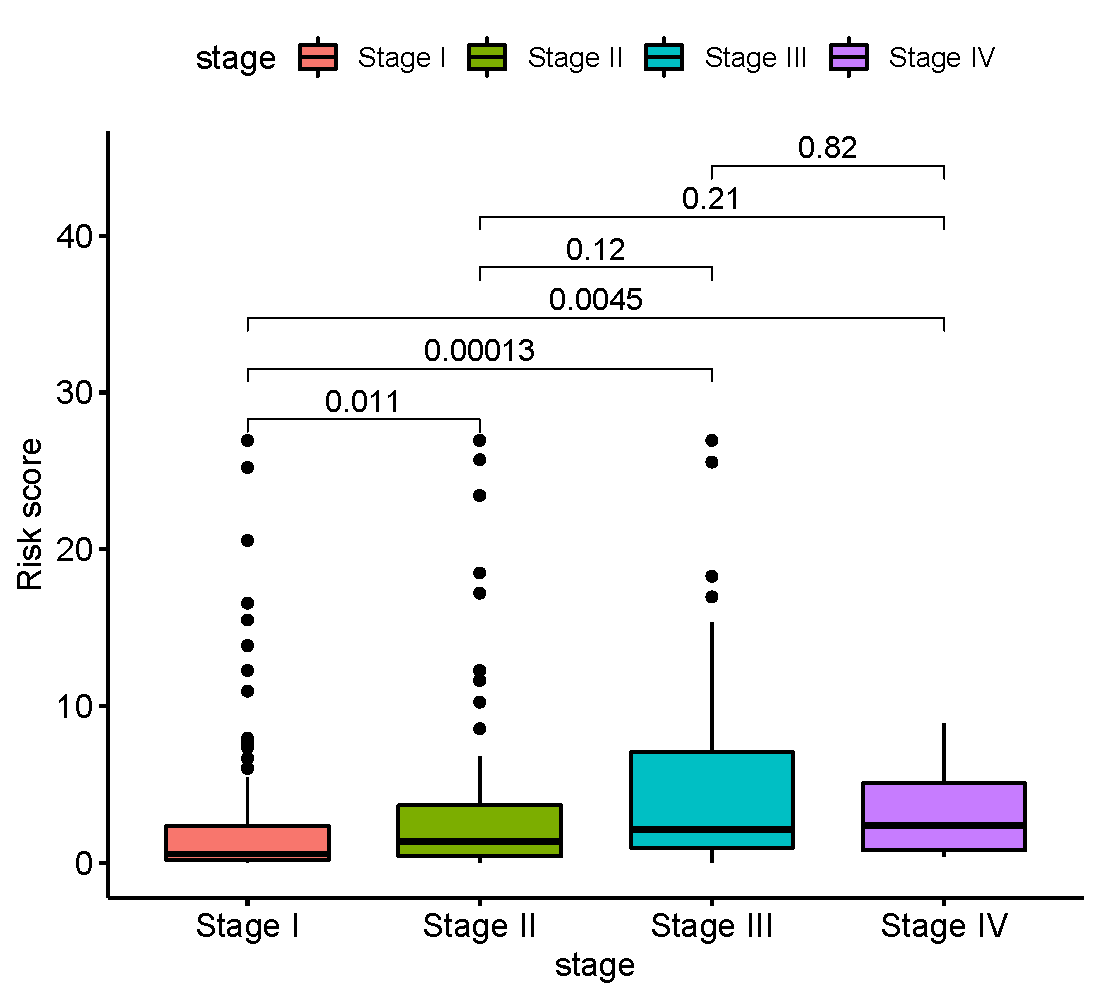

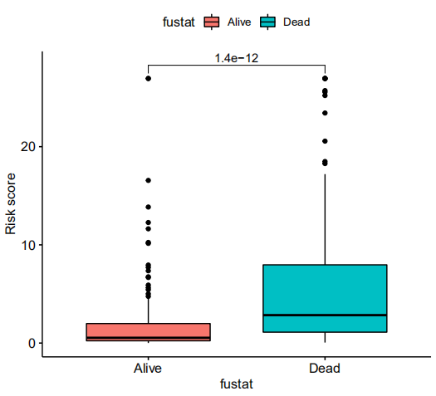


A B


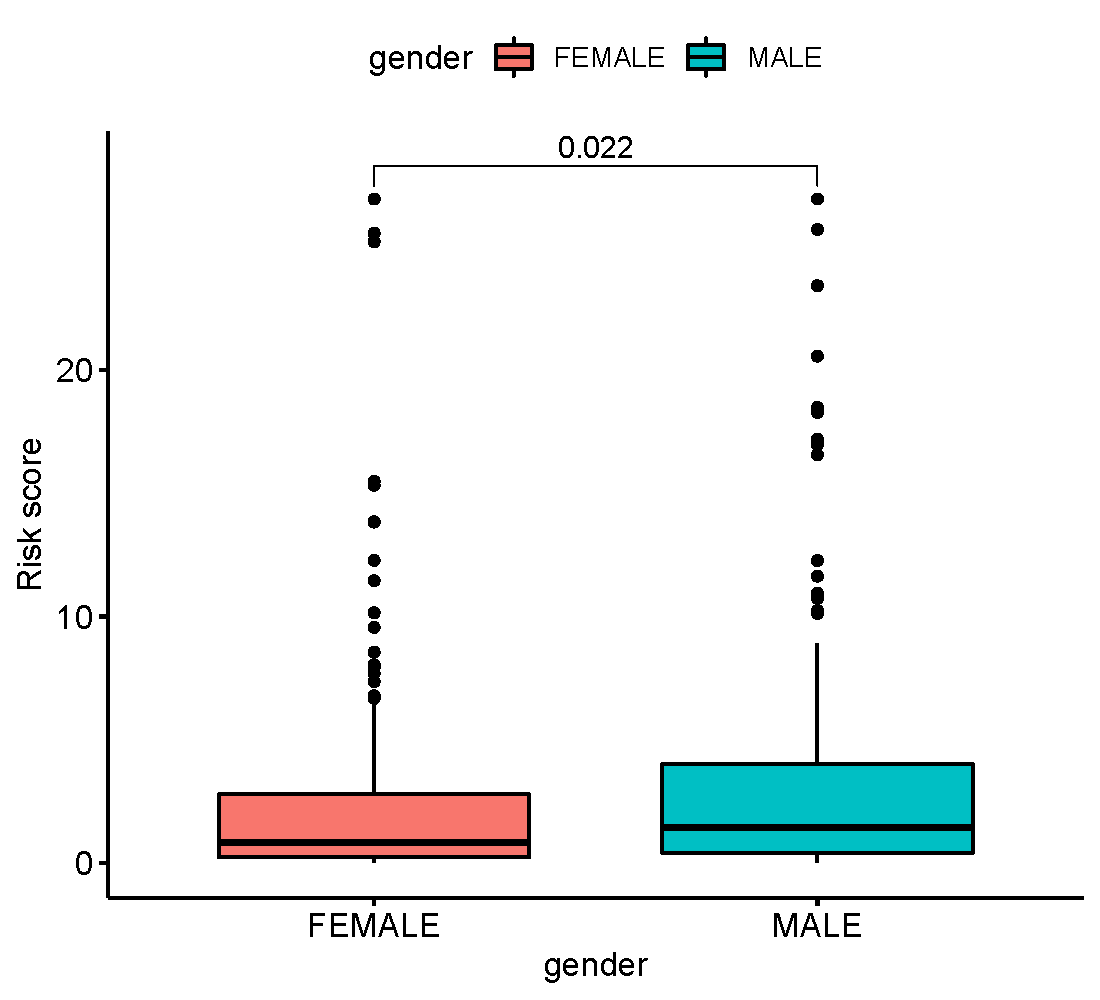

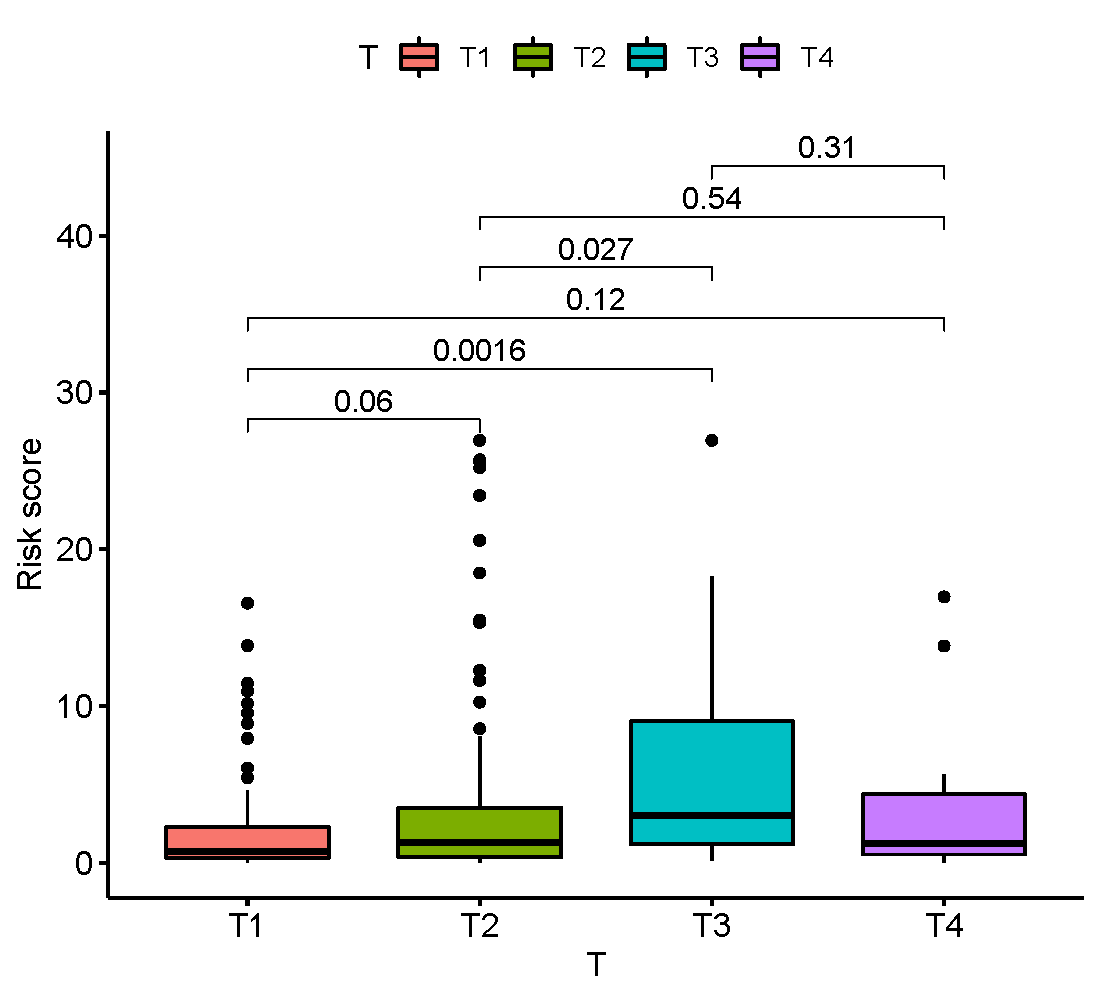
C D


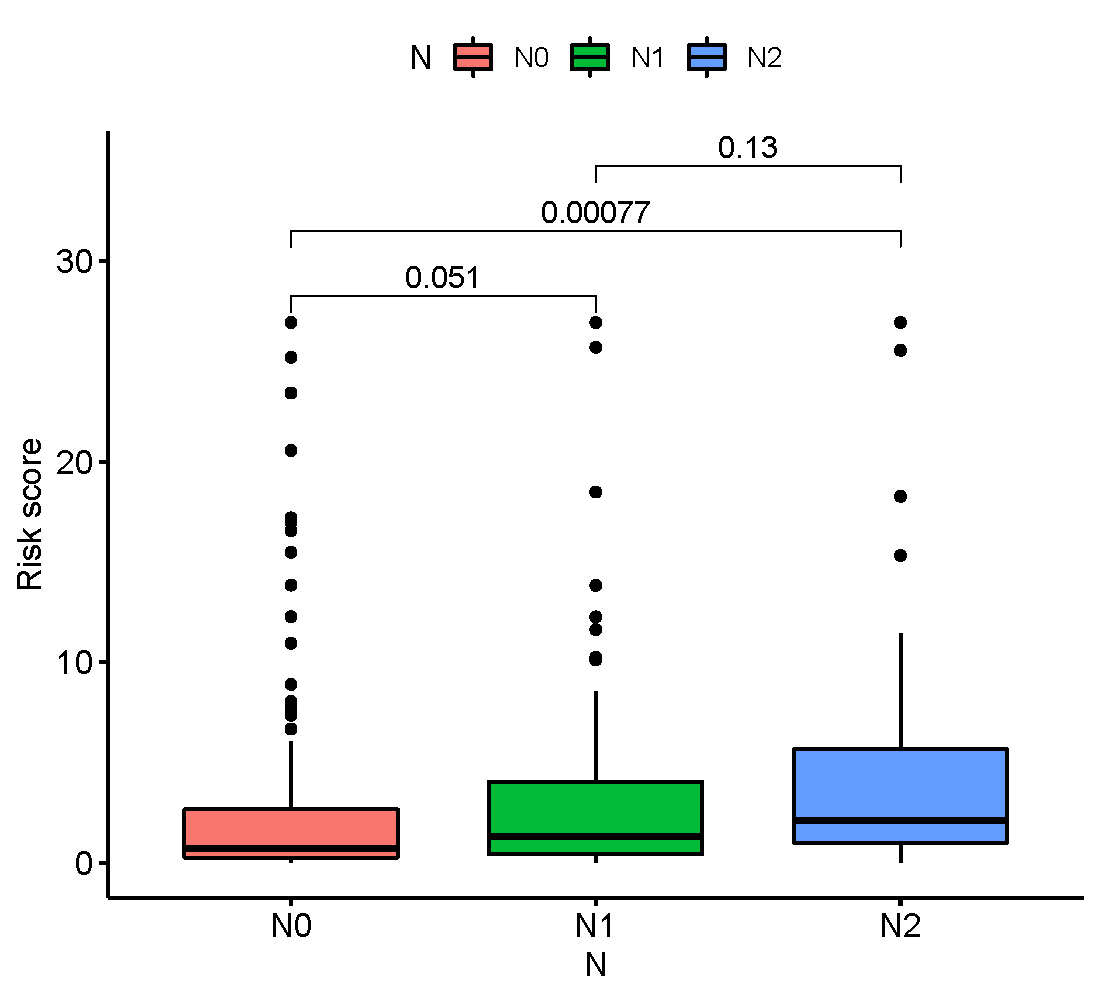

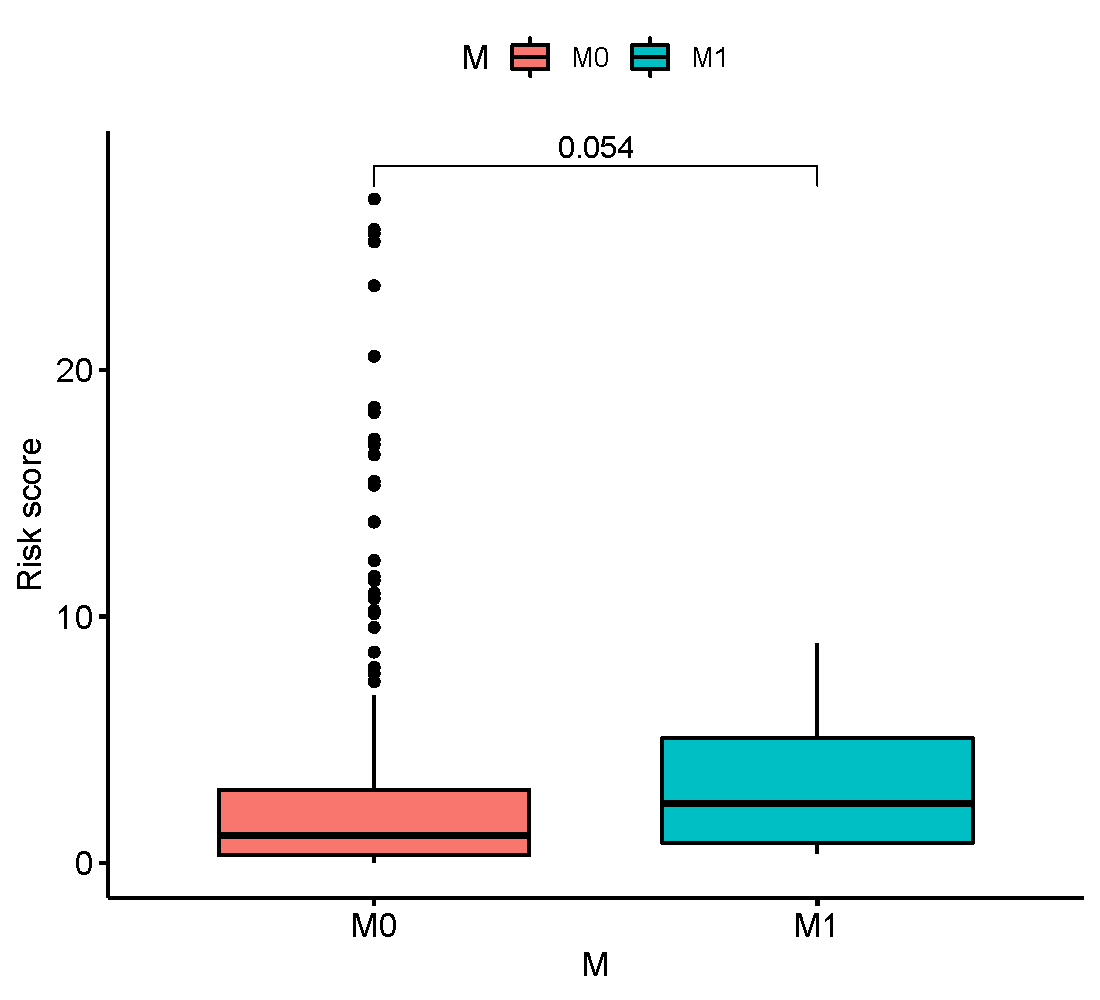
E F

**Fig S2. Correlation between clinicopathological factors and risk score.** The scatter plot shows the correlation between clinicopathological factors and risk scores, including fustat (A), AJCC stage (B), T stage (C), gender (D), M stage (E), and N stage (F).


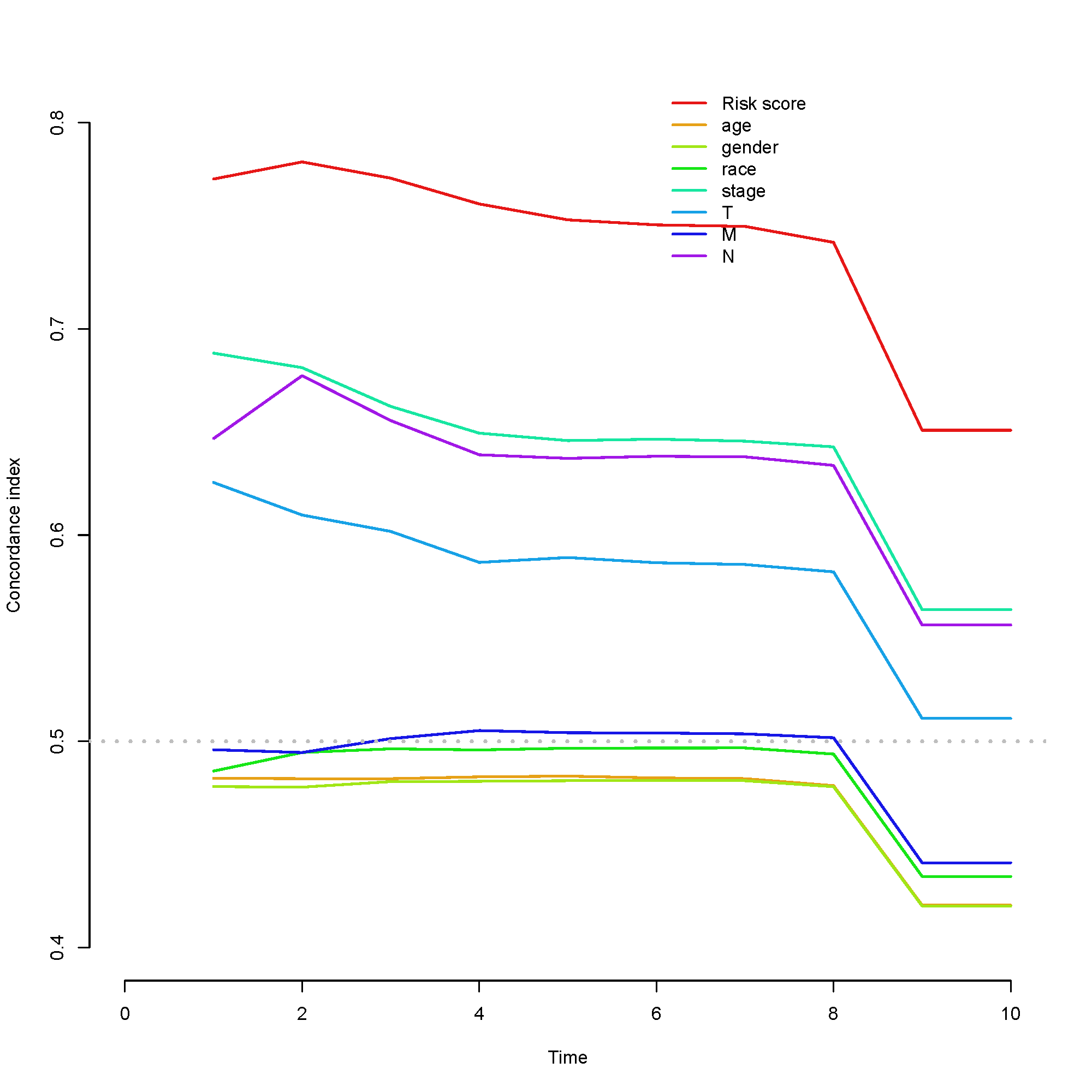
A


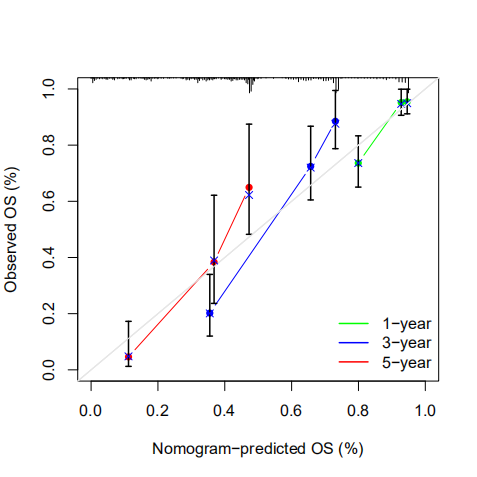


B


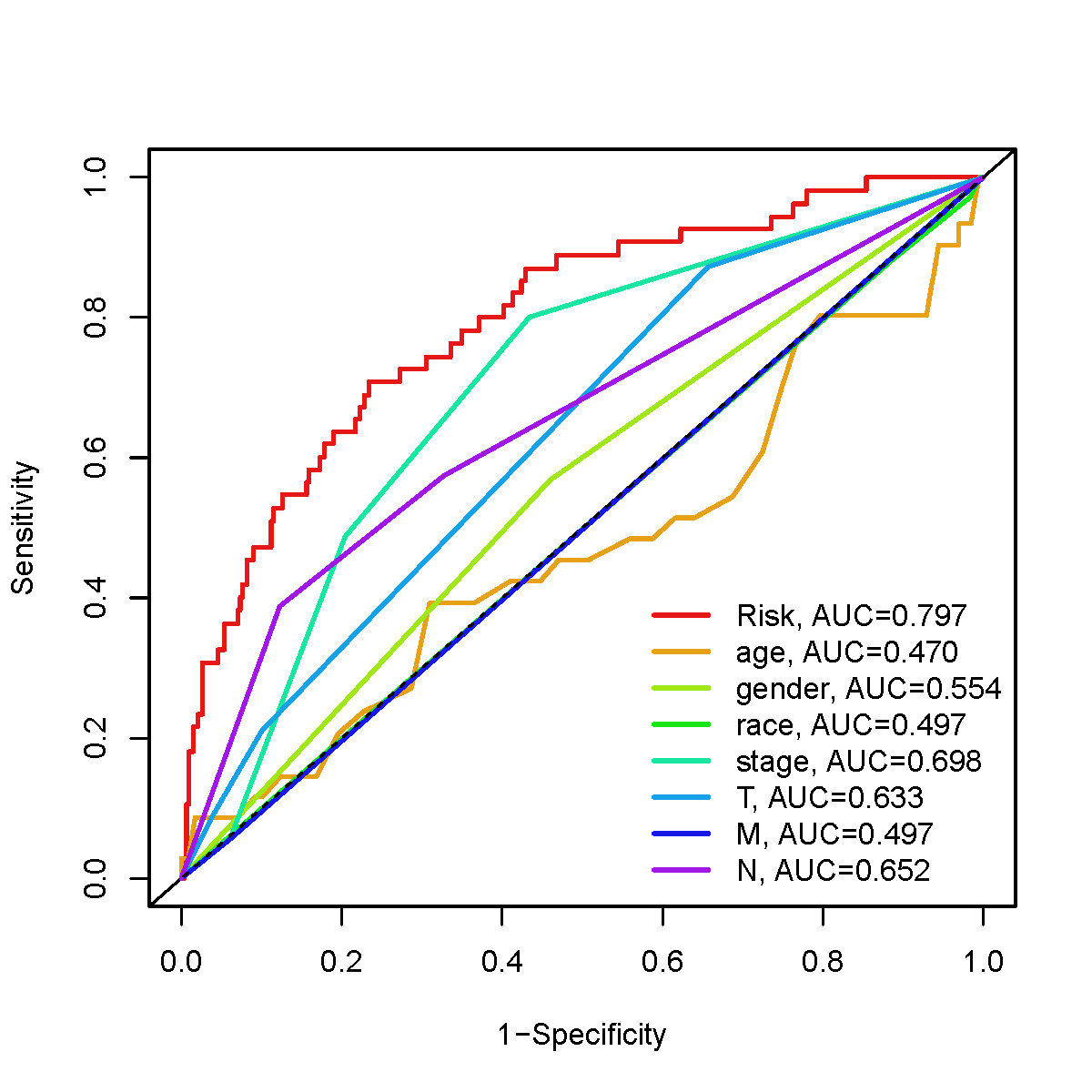

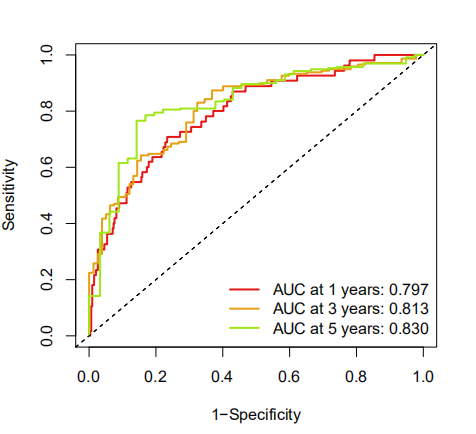
C D


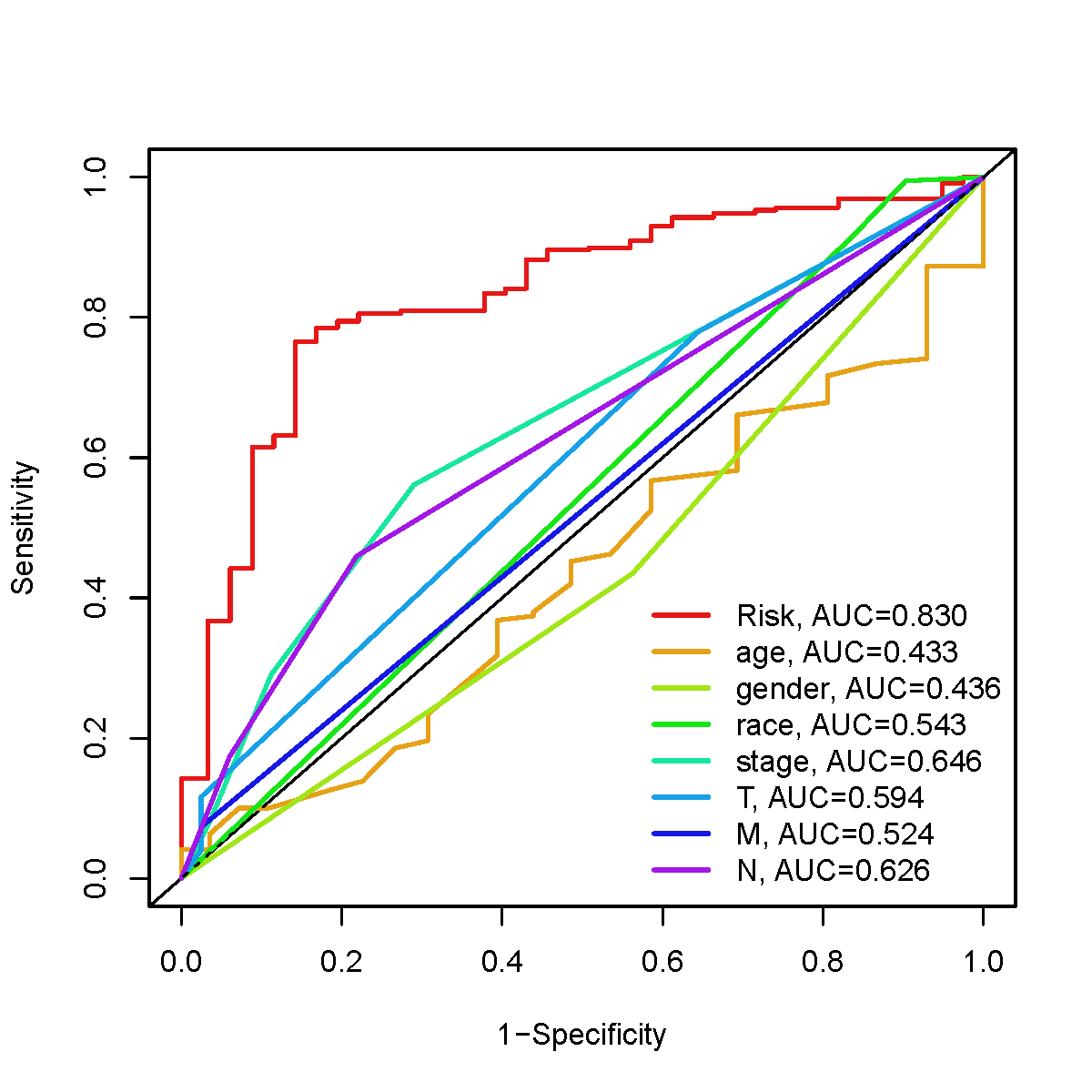

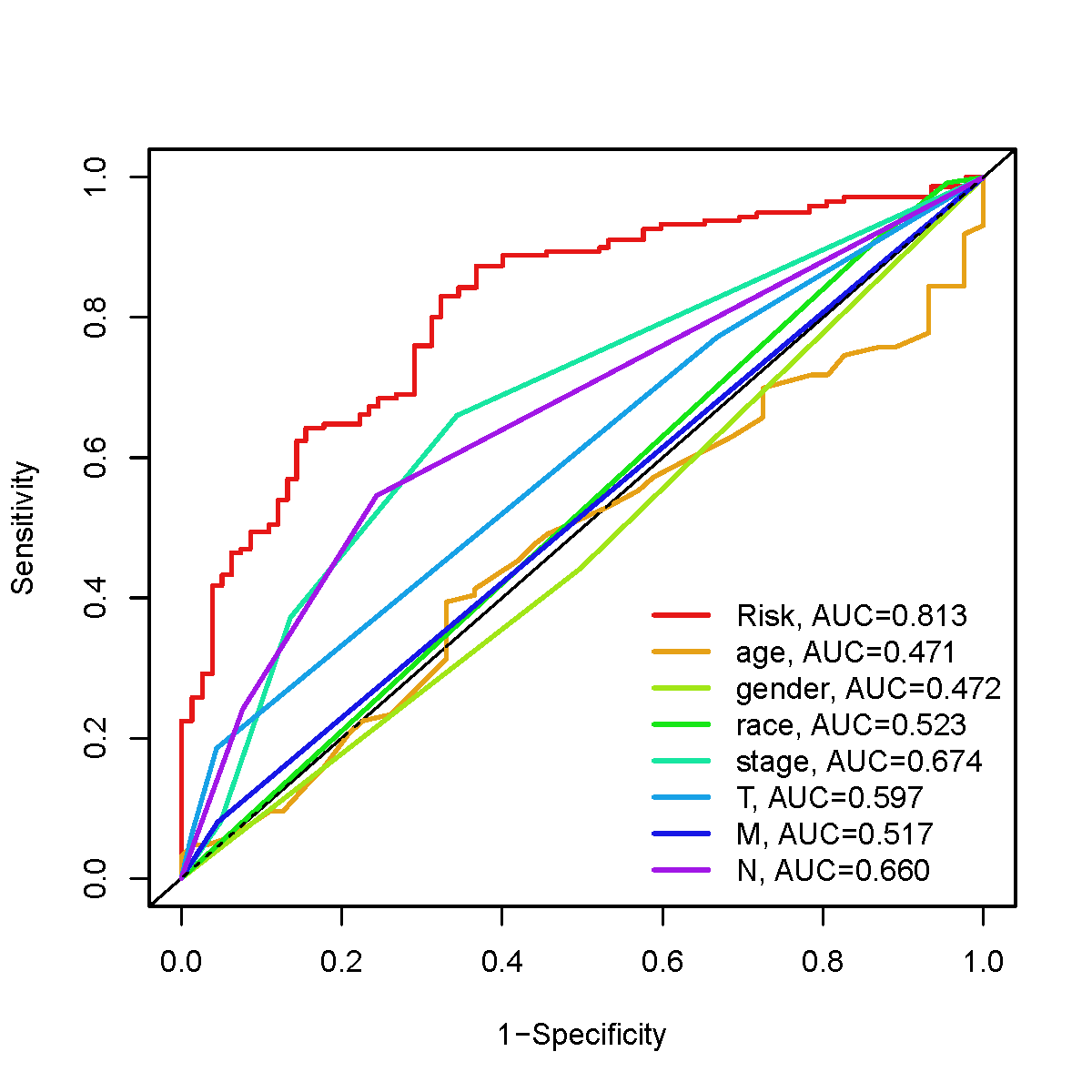
E F

**Fig S3. Survival analysis based on the prognostic model.** According to the C-index method, we verified that multiple indexes were greater than 0.5 (A). Panel B represents the entire cohort survival calibration curve. The white diagonal bar charts represent the ideal. The green, blue, and red bars represent 1-year, 3-year, and 5-year survival, respectively. The model predicts the survival time of patients with good accuracy in the short and long term (1-year AUC=0.797, 3-year AUC=0.813, 5-year AUC=0.830) (C). Most of the indicators in the model, such as risk, age, stage, T, M, and N, can be used as predictors at 1-year (D), 3-year (E), and 5-year (F) survival.


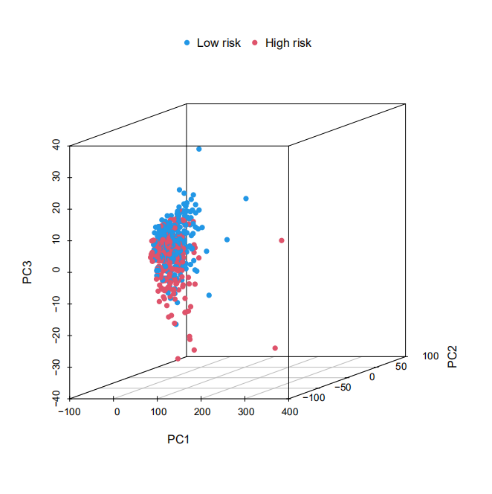

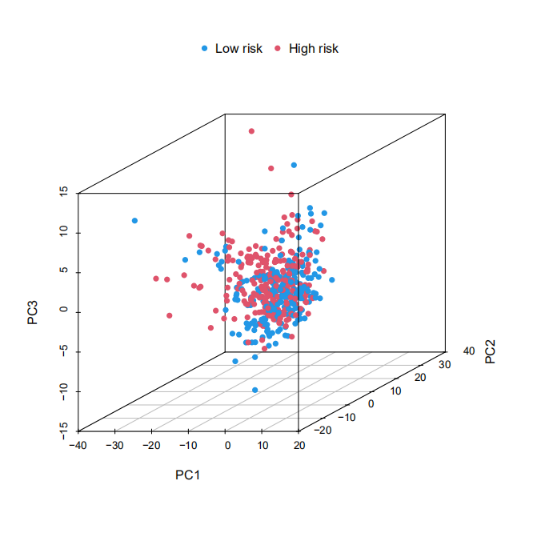
A B


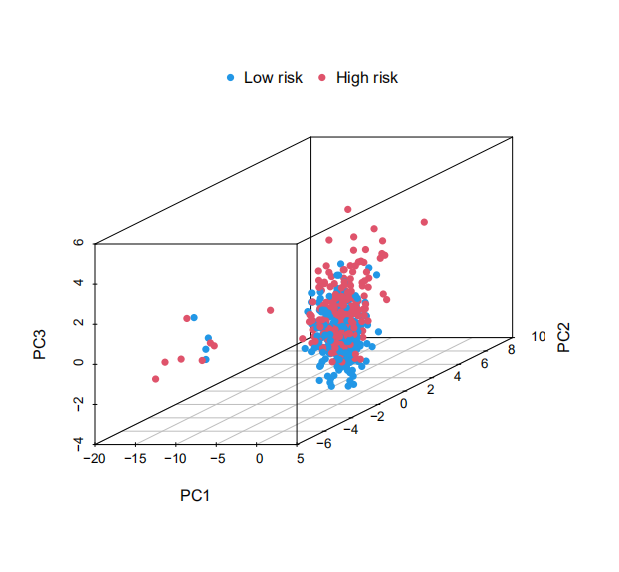


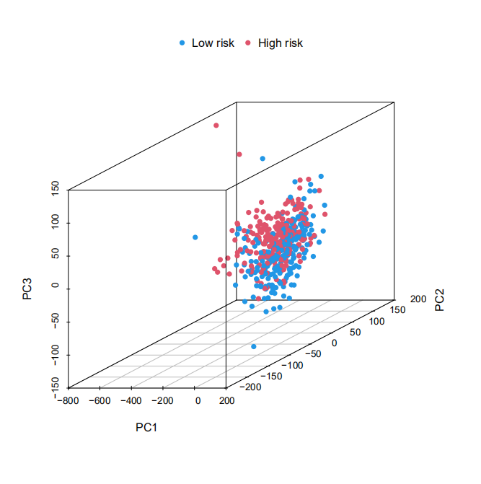
C D

**Fig S4. Principal component analysis of gene expression profiles.** The red dots in the figure were from patients in the high-risk group, and the blue dots were from patients in the low-risk group, indicating the separation of red and blue samples of mRNA (A), lncRNA (B), risk lncRNA (C), and all genes (D) in the risk groups was relatively complete.

A


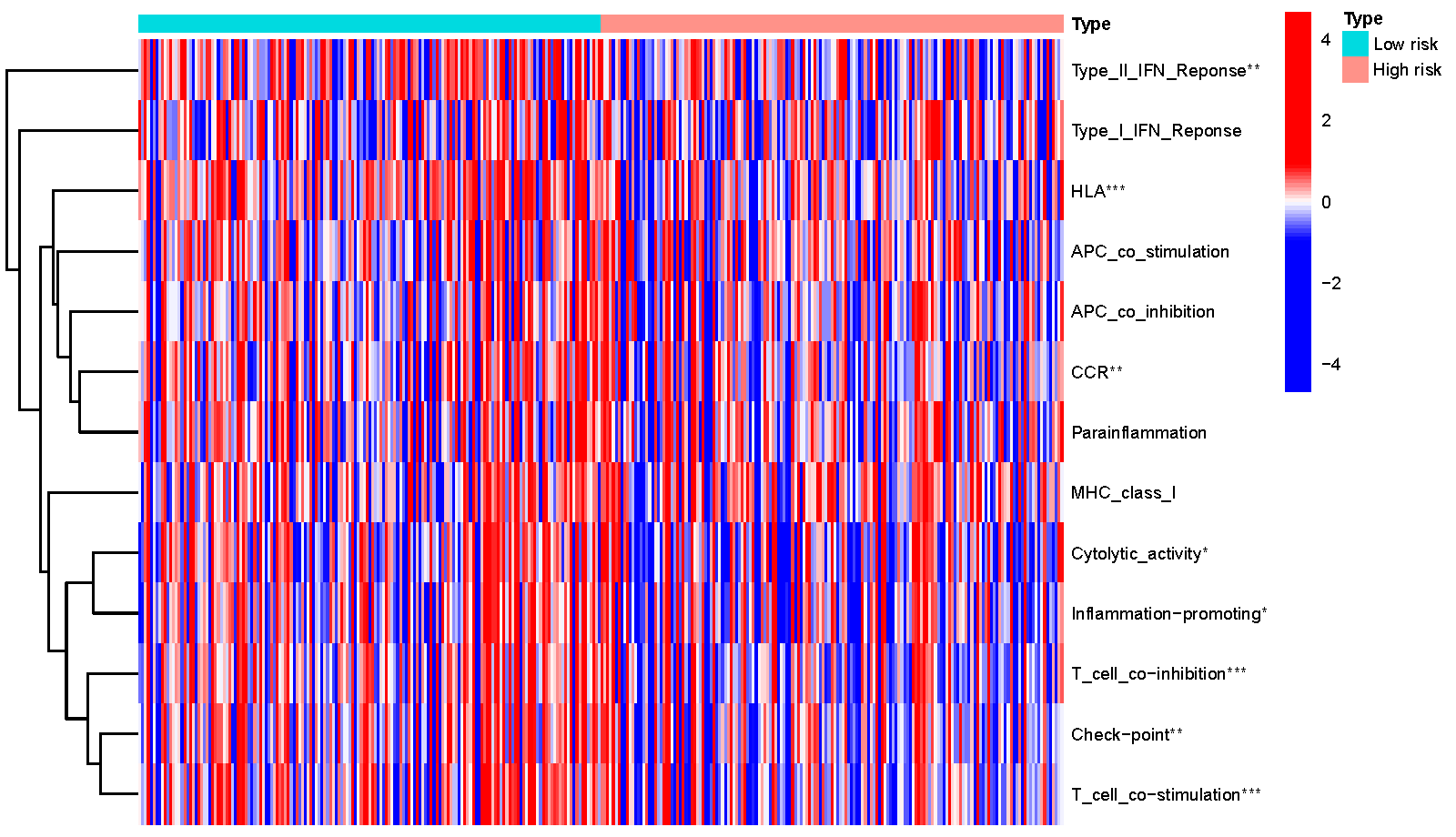


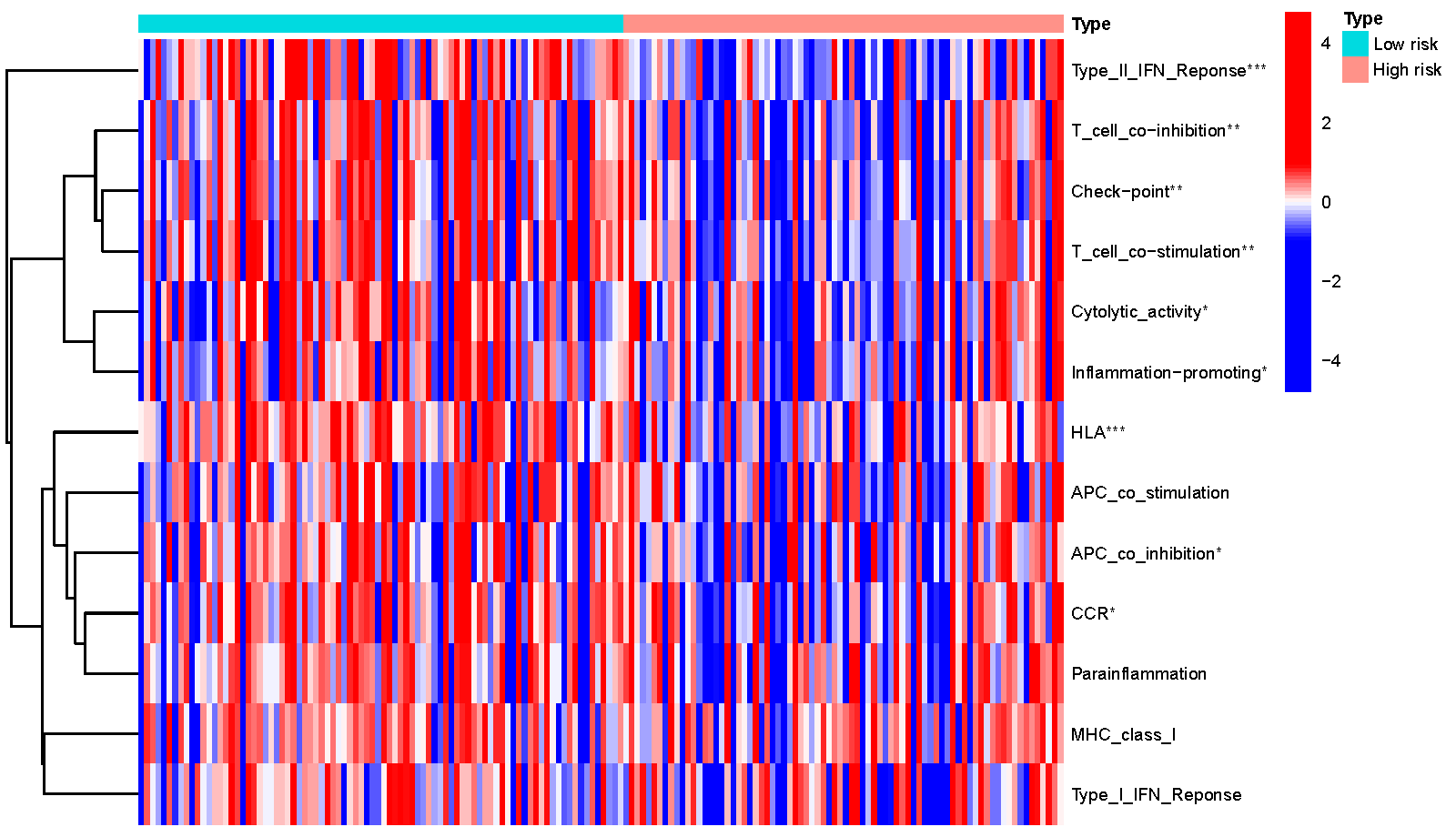
B


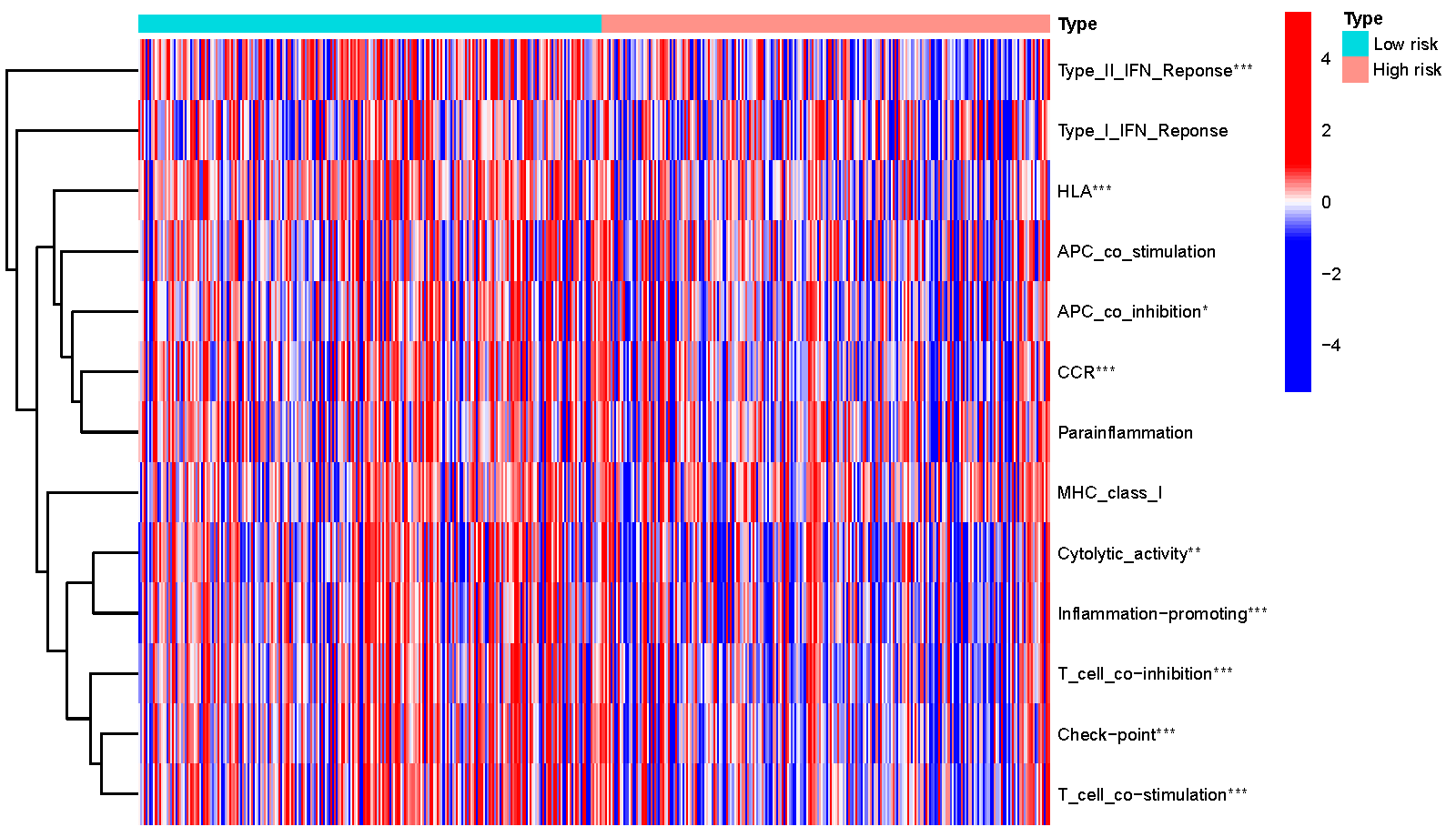
C


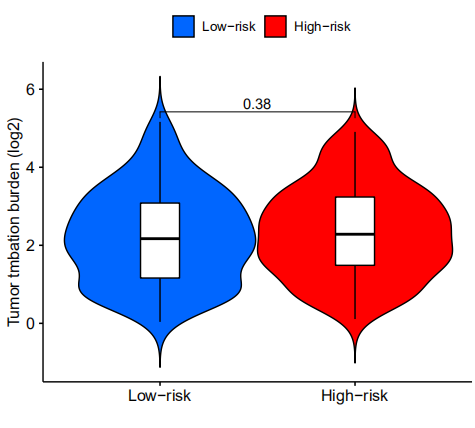
D


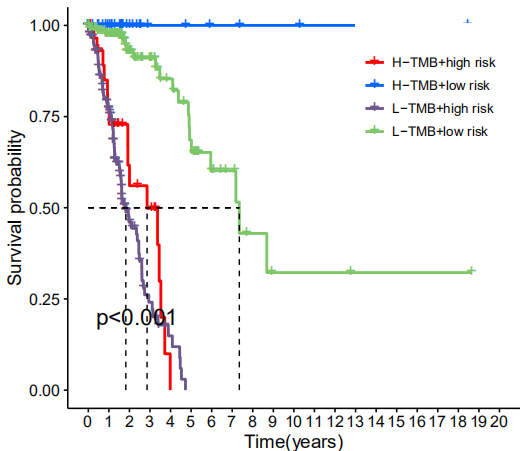
E

**Fig S5. Immune function analysis based on our prognostic model.** In the same immune cluster, the high expression of Cylolytic_activity, Inflammation-promoting, T_cell_co-inhibition, Check-point, T_cell_co-stimulation are significantly related to the low-risk subgroup in the training cohort(A), validation cohort (B) and all cohort (C). Differential analysis of TMB and CELncSigs (D). The high- and low-risk subgroups and high and low TMB were combined to conduct the Kaplan-Meier test (E).


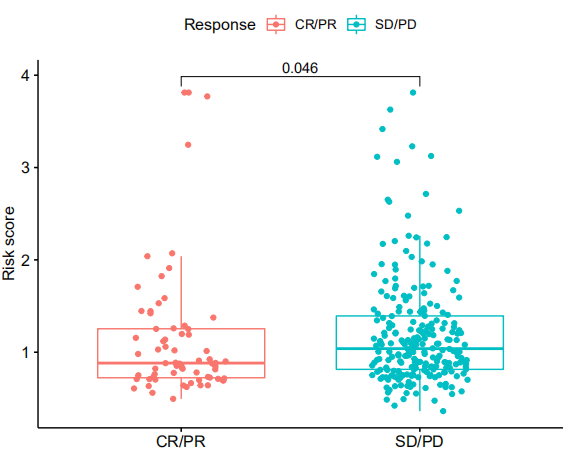

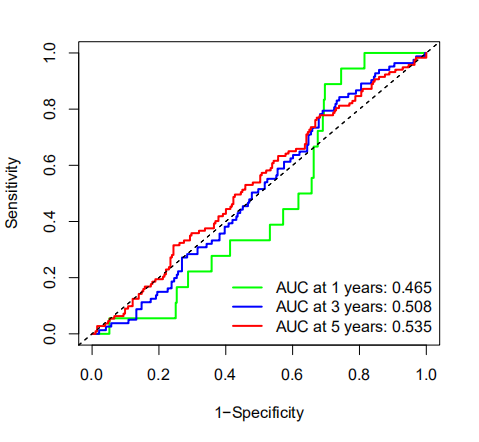


A

B


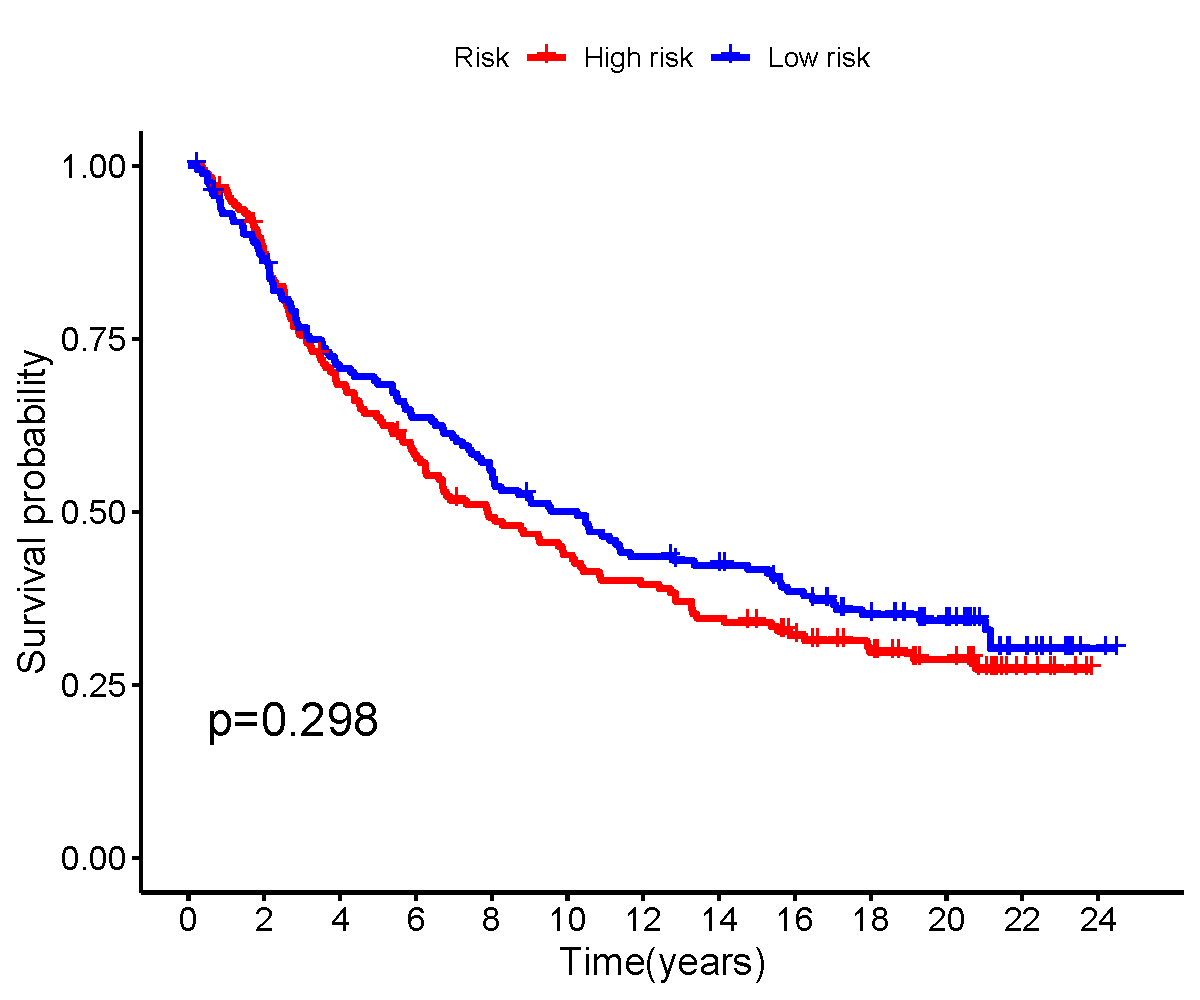
C

**Fig S6. Response to immunotherapy in high- and low-risk groups.** Significant differences were found between responses to different drugs for immunotherapy (A) (P<0.05), where CR is Complete Response, PR is Partial Response, SD is Stable Disease, and PD is Progressive Disease. The immune model is not effective in predicting patient prognosis (B) as reflected in the poor AUC curve (C).


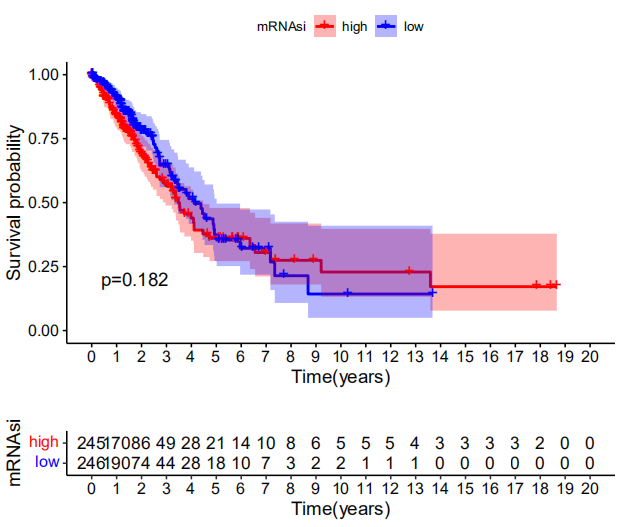


A


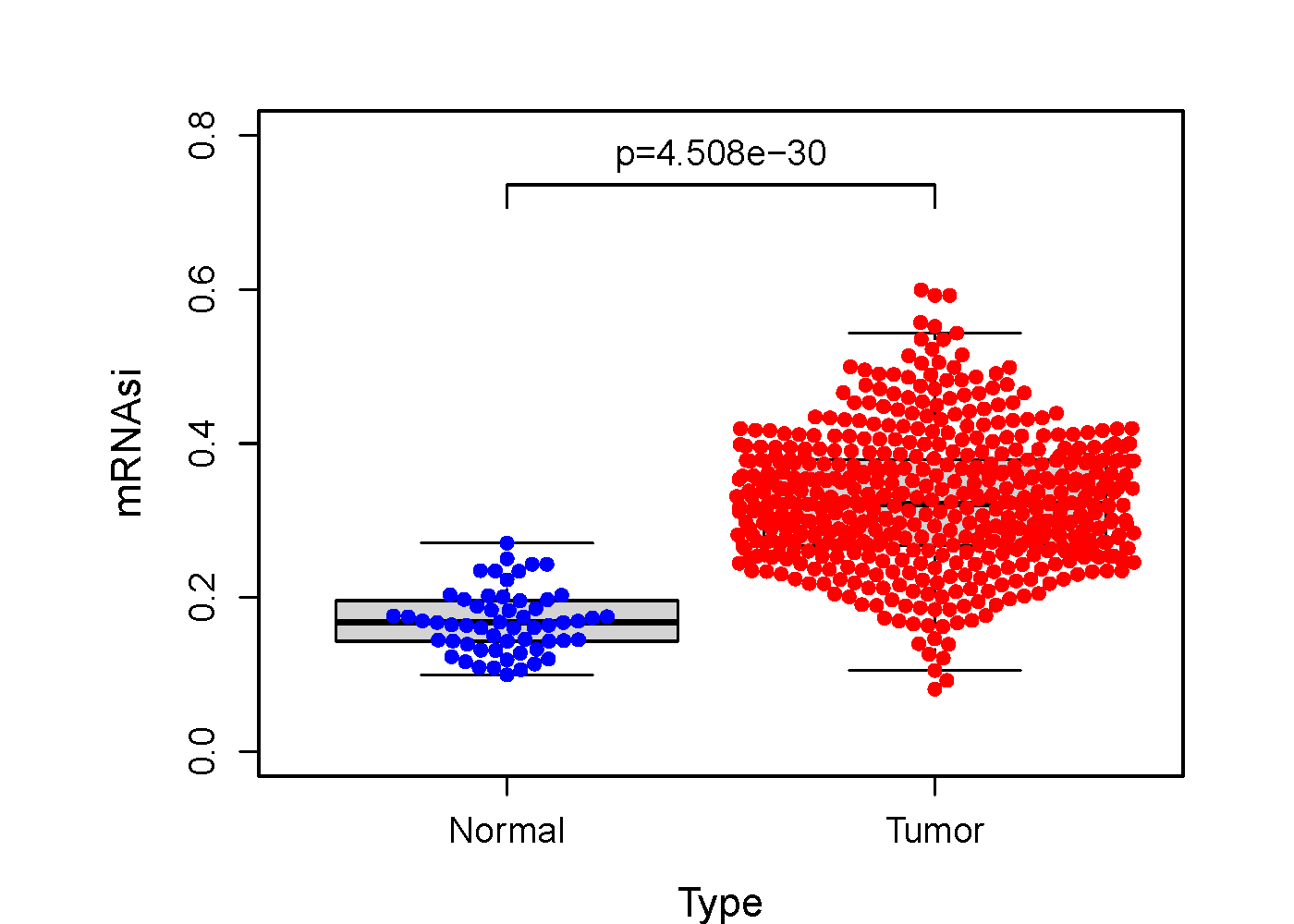


B


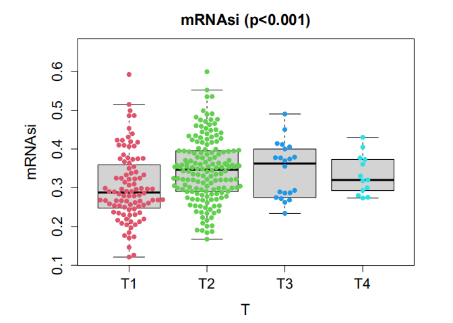

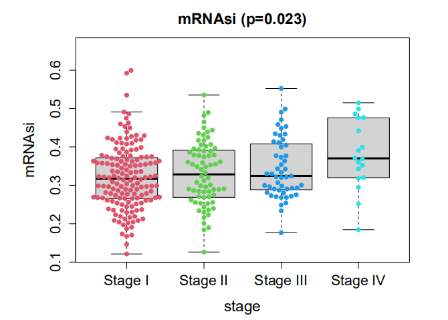


C D

E F


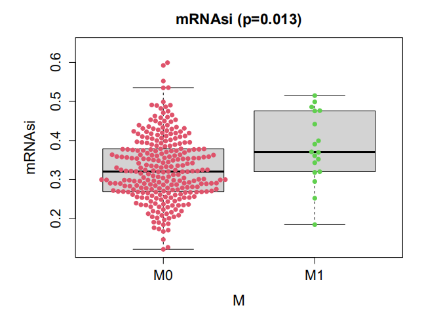

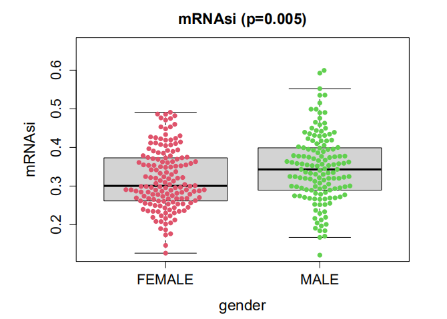


**Fig S7. Comparison of mRNAsi levels between different groups.** No significant difference was found in overall survival between high- and low-risk groups (A). mRNAsi was significantly different between normal and tumor tissues (B), T stage (C), AJCC stage (D), gender (E), and M stage (F).

| **Covariates** | **Total** | **Validation cohort(n=100)** | | **Training cohort(n=194)** | **P-value** |
| --- | --- | --- | --- | --- | --- |
|  | **Number(%)** | | **Number(%)** | **Number(%)** |  |
| **Fustat** |  | |  |  |  |
| **Alive** | **190(64.63%)** | | **64(64%)** | **126(64.95%)** | **0.9742** |
| **Dead** | **104(35.37%)** | | **36(36%)** | **68(35.05%)** |  |
| **Age** |  | |  |  |  |
| **<=65** | **147(50%)** | | **47(47%)** | **100(51.55%)** | **0.5382** |
| **>65** | **147(50%)** | | **53(53%)** | **94(48.45%)** |  |
| **Gender** |  | |  |  |  |
| **Female** | **155(52.72%)** | | **55(55%)** | **100(51.55%)** | **0.6609** |
| **Male** | **139(47.28%)** | | **45(45%)** | **94(48.45%)** |  |
| **Race** |  | |  |  |  |
| **American Indian**  **or Alaska Native** | **1(0.34%)** | | **0(0%)** | **1(0.52%)** | **0.896** |
| **Asian** | **5(1.7%)** | | **2(2%)** | **3(1.55%)** |  |
| **Black or African American** | **27(9.18%)** | | **9(9%)** | **18(9.28%)** |  |
| **White** | **261(88.78%)** | | **89(89%)** | **172(88.66%)** |  |
| **Stage** |  | |  |  |  |
| **Stage I** | **153(52.04%)** | | **50(50%)** | **103(53.09%)** | **0.9268** |
| **Stage II** | **72(24.49%)** | | **26(26%)** | **46(23.71%)** |  |
| **Stage III** | **51(17.35%)** | | **17(17%)** | **34(17.53%)** |  |
| **Stage IV** | **18(6.12%)** | | **7(7%)** | **11(5.67%)** |  |
| **T** |  | |  |  |  |
| **T1** | **96(32.65%)** | | **35(35%)** | **61(31.44%)** | **0.6375** |
| **T2** | **162(55.1%)** | | **51(51%)** | **111(57.22%)** |  |
| **T3** | **23(7.82%)** | | **10(10%)** | **13(6.7%)** |  |
| **T4** | **13(4.42%)** | | **4(4%)** | **9(4.64%)** |  |
| **M** |  | |  |  |  |
| **M0** | **276(93.88%)** | | **93(93%)** | **183(94.33%)** | **0.8463** |
| **M1** | **18(6.12%)** | | **7(7%)** | **11(5.67%)** |  |
| **N** |  | |  |  |  |
| **N0** | **189(64.29%)** | | **67(67%)** | **122(62.89%)** | **0.3866** |
| **N1** | **60(20.41%)** | | **16(16%)** | **44(22.68%)** |  |
| **N2** | **45(15.31%)** | | **17(17%)** | **28(14.43%)** |  |
|  |  | |  |  |  |

**Table S1. The classification of clinical data.** P value >0.05 reflects no statistical difference between the training and validation cohorts, indicating that the grouping results are promising.

| **LncRNA** | **Coefficient** | **HR** | **HR.95L** | | **HR.95H** | | **Pvalue** |
| --- | --- | --- | --- | --- | --- | --- | --- |
| **Z97989.1** | **-0.59763** | **0.550112** | **0.274656** | **1.101825** | | **0.091729** | |
| **AC022165.1** | **0.908828** | **2.481412** | **1.230256** | **5.004981** | | **0.011122** | |
| **AC008937.3** | **-0.59229** | **0.553057** | **0.25551** | **1.197104** | | **0.132752** | |
| **FAM30A** | **-0.22901** | **0.795324** | **0.586599** | **1.078319** | | **0.140355** | |
| **AC138965.1** | **1.227433** | **3.412459** | **1.948889** | **5.975137** | | **1.75E-05** | |
| **AC026355.2** | **-0.39424** | **0.67419** | **0.537416** | **0.845774** | | **0.000655** | |
| **AP001107.5** | **0.871848** | **2.391325** | **1.283522** | **4.455271** | | **0.006029** | |
| **`NHS-AS1`** | **0.880603** | **2.412353** | **1.34423** | **4.329204** | | **0.003163** | |
| **`RTCA-AS1`** | **-0.43023** | **0.650357** | **0.435754** | **0.97065** | | **0.035225** | |
| **AL008723.2** | **0.460493** | **1.584855** | **1.104301** | **2.274529** | | **0.012483** | |
| **AC021087.3** | **-0.84525** | **0.429449** | **0.232098** | **0.794605** | | **0.007097** | |
| **AL355472.3** | **0.385346** | **1.470123** | **1.001369** | **2.158307** | | **0.049189** | |
| **CYTOR** | **0.231717** | **1.260763** | **0.940379** | **1.690301** | | **0.121377** | |
| **AL031600.2** | **-1.75188** | **0.173448** | **0.073423** | **0.409738** | | **6.49E-05** | |
| **AL162632.3** | **1.249339** | **3.488038** | **1.300728** | **9.353538** | | **0.013051** | |
| **AC105020.5** | **-0.63033** | **0.532414** | **0.305468** | **0.927968** | | **0.026169** | |
| **AL512413.1** | **0.239128** | **1.270141** | **0.998694** | **1.615368** | | **0.051259** | |
| **LINC01138** | **0.378575** | **1.460203** | **1.048319** | **2.033915** | | **0.025152** | |
| **AC012409.4** | **0.851599** | **2.343391** | **1.199702** | **4.577373** | | **0.012668** | |
| **AC007663.4** | **-0.46135** | **0.630432** | **0.391306** | **1.015686** | | **0.05796** | |
| **`FLG-AS1`** | **0.489495** | **1.631492** | **0.971221** | **2.740639** | | **0.064368** | |
| **AC092794.2** | **1.137985** | **3.120476** | **1.647688** | **5.909717** | | **0.000478** | |
| **AC007686.2** | **-1.80873** | **0.163862** | **0.035725** | **0.751594** | | **0.019943** | |
| **AC006449.5** | **0.449369** | **1.567323** | **1.075836** | **2.283343** | | **0.019246** | |
| **OGFRP1** | **0.793982** | **2.212188** | **1.456027** | **3.361048** | | **0.000199** | |

**Table S2.** **25 lncRNAs associated with chromatin epigenetics in the risk model.**
